# An EFR‐Cf‐9 chimera confers enhanced resistance to bacterial pathogens by SOBIR1‐ and BAK1‐dependent recognition of elf18

**DOI:** 10.1111/mpp.12789

**Published:** 2019-04-01

**Authors:** Jinbin Wu, Ida‐Barbara Reca, Francesco Spinelli, Damiano Lironi, Giulia De Lorenzo, Palmiro Poltronieri, Felice Cervone, Matthieu H.A.J. Joosten, Simone Ferrari, Alexandre Brutus

**Affiliations:** ^1^ Laboratory of Phytopathology Wageningen University Droevendaalsesteeg 1 6708 PB Wageningen Netherlands; ^2^ CNR‐ISPA, via Provinciale Lecce‐Monteroni 73100 Lecce Italy; ^3^ Department of Biology and Biotechnology “Charles Darwin” Sapienza University of Rome 00185 Rome Italy; ^4^ DOE Plant Research Laboratory Michigan State University East Lansing MI 48824 USA

**Keywords:** BAK1, Cf‐9, EFR, pattern recognition receptors, plant innate immunity, R genes, SOBIR1

## Abstract

The transfer of well‐studied native and chimeric pattern recognition receptors (PRRs) to susceptible plants is a proven strategy to improve host resistance. In most cases, the ectodomain determines PRR recognition specificity, while the endodomain determines the intensity of the immune response. Here we report the generation and characterization of the chimeric receptor EFR‐Cf‐9, which carries the ectodomain of the *Arabidopsis thaliana *EF‐Tu receptor (EFR) and the endodomain of the tomato Cf‐9 resistance protein. Both transient and stable expression of *EFR‐Cf‐9* triggered a robust hypersensitive response (HR) upon elf18 treatment in tobacco. Co‐immunoprecipitation and virus‐induced gene silencing studies showed that EFR‐Cf‐9 constitutively interacts with SUPPRESSOR OF BIR1‐1 (SOBIR1) co‐receptor, and requires both SOBIR1 and kinase‐active BRI1‐ASSOCIATED KINASE1 (BAK1) for its function. Transgenic plants expressing *EFR‐Cf‐9* were more resistant to the (hemi)biotrophic bacterial pathogens *Pseudomonas amygdali *pv. *tabaci* (*Pta*) 11528 and *Pseudomonas syringae *pv. *tomato* DC3000, and mounted an HR in response to high doses of *Pta *11528 and *P. carotovorum*. Taken together, these data indicate that the EFR‐Cf‐9 chimera is a valuable tool for both investigating the molecular mechanisms responsible for the activation of defence responses by PRRs, and for potential biotechnological use to improve crop disease resistance.

## Introduction

Plants have evolved an innate immune system that relies on the recognition of potential pathogens by a defined pool of membrane and cytosolic receptors. The first layer of plant immunity comprises plasma membrane pattern recognition receptors (PRRs) that recognize microbe‐associated molecular patterns (MAMPs), thereby mounting MAMP‐triggered immunity (MTI) (Cook *et al*., 2015; Couto and Zipfel, 2016; Dangl *et al*., 2013; Dodds and Rathjen, 2010). Successful pathogens often produce effector proteins capable of suppressing MTI and preventing an effective immune response. As a countermeasure, plants have evolved pathogen strain‐specific immune receptors, so‐called resistance (R) proteins, which are capable of recognizing particular effectors and subsequently activate effector‐triggered immunity (ETI). Although leading to outputs that are qualitatively similar to those of MTI, ETI is generally stronger and faster, and often includes a rapid programmed cell death (PCD) of the host cells at the site of infection, referred to as hypersensitive response (HR) (Chisholm *et al*., 2006; Jones and Dangl, 2006).

Cell surface PRRs and R proteins include transmembrane (TM)‐associated receptor‐like kinases (RLKs) and receptor‐like proteins (RLPs), of which the latter lack a cytoplasmic kinase domain. Both types of receptors possess an ectodomain for ligand recognition that in many cases is mainly composed of leucine‐rich repeats (LRRs) (Boutrot and Zipfel, 2017; Zipfel, 2014). In tomato (*Solanum lycopersicum, Sl*), the well‐studied RLP Cf‐4 triggers a strong HR‐associated immunity against the biotrophic pathogenic fungus *Cladosporium fulvum* secreting the effector Avr4 (Joosten *et al*., 1997; Thomas *et al*., 1997). Cf‐4 shares identical TM and cytoplasmic domains with Cf‐9 (Thomas *et al*., 1997), which confers immunity to *C. fulvum *secreting the Avr9 effector (van Kan *et al*., 1991). The RLK SUPPRESSOR OF BIR1‐1/EVERSHED (SOBIR1/EVR, hereafter referred to as SOBIR1) is required for Cf‐4‐mediated resistance to *C. fulvum *(Liebrand *et al*., 2013) and, through its TM domain, constitutively interacts with Cf‐4 (Bi *et al*., 2016). Increasing evidence suggests that SOBIR1 constitutively interacts with several RLPs, providing the RLP/SOBIR1 complex with a kinase domain to trigger downstream signalling pathways (Albert *et al*., 2015; Böhm *et al*., 2014; Domazakis *et al*., 2018; Gust and Felix, 2014; Hegenauer *et al*., 2016; Liebrand *et al*., 2013, 2014; Ma and Borhan, 2015; Wang *et al.*, 2018; Zhang *et al*., 2013, 2014). An additional RLK, BAK1, the orthologue of the *Arabidopsis thaliana* (*At*) BRI‐ASSOCIATED RECEPTOR KINASE 1/SOMATIC EMBRYOGENESIS RECEPTOR KINASE 3 (BAK1/SERK3, hereafter referred to as *At*BAK1), is recruited to the Cf‐4/SOBIR1 complex after perception of Avr4 by Cf‐4 and is required for Cf‐4‐mediated resistance (Postma *et al*., 2016).

Elf18, an 18‐amino acid N‐terminal epitope of the bacterial elongation factor Tu (EF‐Tu) (Kunze *et al*., 2004), is recognized by the Arabidopsis LRR‐RLK EF‐Tu receptor (EFR) and triggers MTI (Zipfel *et al*., 2006). The ‘prototypical’ elf18 peptide from *Escherichia coli *(MSKEKFERTKPHVNVGTI; elf18D, hereafter referred to as elf18) and those from *Erwinia amylovora *and* Erwinia chrysanthemi *are identical and show full activity, whereas elf18 peptides with slightly varying sequences, as found in additional bacteria, show different activity (Kunze *et al*., 2004). For example, elf18C from *Agrobacterium *strains and elf18F, shared by different *Pseudomonas* species, including *Pseudomonas amygdali *pv. *tabaci *(*Pta*) 11528, are fully active, whereas elf18B and elf18G, found in *Xanthomonas campestris *pv. *campestris *(*Xcc*) B100 and *Pseudomonas syringae *pv. *tomato* (*Pst*) DC3000, respectively, trigger a weaker response (Lacombe *et al*., 2010). Besides elf18, a widely studied MAMP is flg22, an epitope of the bacterial flagellin that is recognized by the LRR‐RLK FLAGELLIN SENSITIVE2 (FLS2) (Felix *et al*., 1999; Gómez‐Gómez and Boller, 2000).

Interfamily transfer of PRRs is an effective strategy for improving resistance of plants to a wide range of infectious microbes (Dangl *et al*., 2013). For example, EFR confers broad‐spectrum resistance to bacterial pathogens in tomato and *Nicotiana benthamiana *(*Nb*) (Lacombe *et al*., 2010), and also in the monocot rice (Schwessinger *et al*., 2015). The rice resistance protein Xa21, which confers resistance to *Pst* DC3000 upon transfer to Arabidopsis (Holton *et al*., 2015), has also been transferred to crops like citrus (Mendes *et al*., 2010), tomato (Afroz *et al*., 2011) and banana (Tripathi *et al*., 2014), to confer resistance to *Xanthomonas *species. Strategies based on stacking multiple resistance genes are also pursued to avoid that the *R* gene‐mediated resistance might be overcome by pathogens. For example, transgenic potato (*Solanum tuberosum*) plants expressing three *R* genes against *Phytophthora infestans* show robust resistance (Chen and Ow, 2017; Halpin, 2005; Zhu *et al*., 2012).

Recent research provides evidence that engineering novel recombinant PRRs by domain swapping to obtain chimeric receptors that combine useful features of different PRRs is a promising option to breed for durable and wide spectrum resistance (Boutrot and Zipfel, 2017; De Lorenzo *et al*., 2011). Significant progress has been achieved, leading to the indication that the ectodomain of a chimeric receptor retains the ligand perception ability, while the endodomain maintains the output intensity (Albert *et al*., 2010; Brutus *et al*., 2010; He *et al*., 2000; Holton *et al*., 2015; Kishimoto *et al*., 2010; Kouzai *et al*., 2013; Schwessinger *et al*., 2015; Thomas *et al*., 2017). For instance, we previously reported that the chimera FLS2‐EFR, carrying the ectodomain of FLS2 and the kinase domain of EFR, recognizes flg22 and induces an EFR‐like response in *N. tabacum* (*Nt, *tobacco), which lacks an endogenous EFR receptor (Lacombe *et al*., 2010). This response typically results in a higher ethylene production than that induced by FLS2 upon perception of flg22 (Brutus *et al*., 2010). WALL‐ASSOCIATED KINASE 1 (WAK1) is a receptor of oligogalacturonides (OGs) and triggers defence responses upon perception of this so‐called damage‐associated molecular pattern (DAMP). Upon OG treatment, a chimera comprising the ectodomain of WAK1 and the EFR kinase domain triggers an EFR‐like response (Brutus *et al*., 2010). On the other hand, a reciprocal EFR‐WAK1 chimera recognizes elf18 to induce a stronger OG/WAK1‐like oxidative burst response than that triggered by EFR upon perception of elf18 (Brutus *et al*., 2010). However, the downstream signalling components employed by the transferred PRRs and chimeras are largely unknown, and the signalling pathways that are triggered in the plant from which the PRR originates and those in the recipient plant might not be conserved.

In this work, we aimed to obtain a MAMP‐dependent robust resistance response in tobacco, by exploiting the EFR‐mediated recognition of the broad‐spectrum bacterial MAMP elf18 and the ability of Cf‐9 to trigger a fast HR upon activation (Hammond‐Kosack *et al*., 1998; Stergiopoulos and de Wit, 2009). Tobacco SR1 plants transiently expressing the chimera EFR‐Cf‐9, harbouring the ectodomain of EFR and the TM and cytoplasmic domain of Cf‐9 (hereafter referred to as the endodomain), indeed mount a strong elf18‐triggered HR. We show that chimeric EFR‐Cf‐9 protein constitutively interacts with SOBIR1, and requires both SOBIR1 and BAK1 for functionality. Transgenic tobacco plants expressing *EFR‐Cf‐9 *activate an HR upon elf18 treatment and show enhanced resistance to the (hemi)biotrophic bacterial pathogens *Pta* 11528 and *Pst* DC3000.

## Results

### EFR‐Cf‐9 recognizes elf18C to trigger a strong HR in tobacco plants

**Figure 1:**
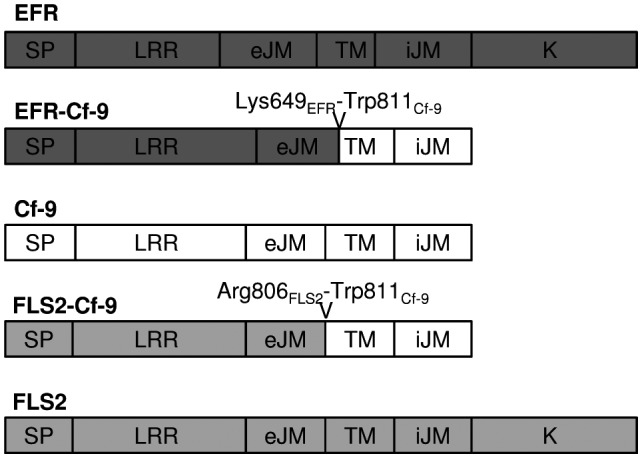
Overview of the structure of the chimeric receptors. The coding regions of EFR, Cf‐9 and FLS2 are indicated in dark grey, white and light grey, respectively. The arrows indicate the junction between the EFR or FLS2 ectodomain and the Cf‐9 transmembrane (TM) domain, with the corresponding residues included in the chimeras. SP, signal peptide; LRR, leucine‐rich repeat; eJM, external juxtamembrane; iJM, internal juxtamembrane; K, kinase.

**Figure 2:**
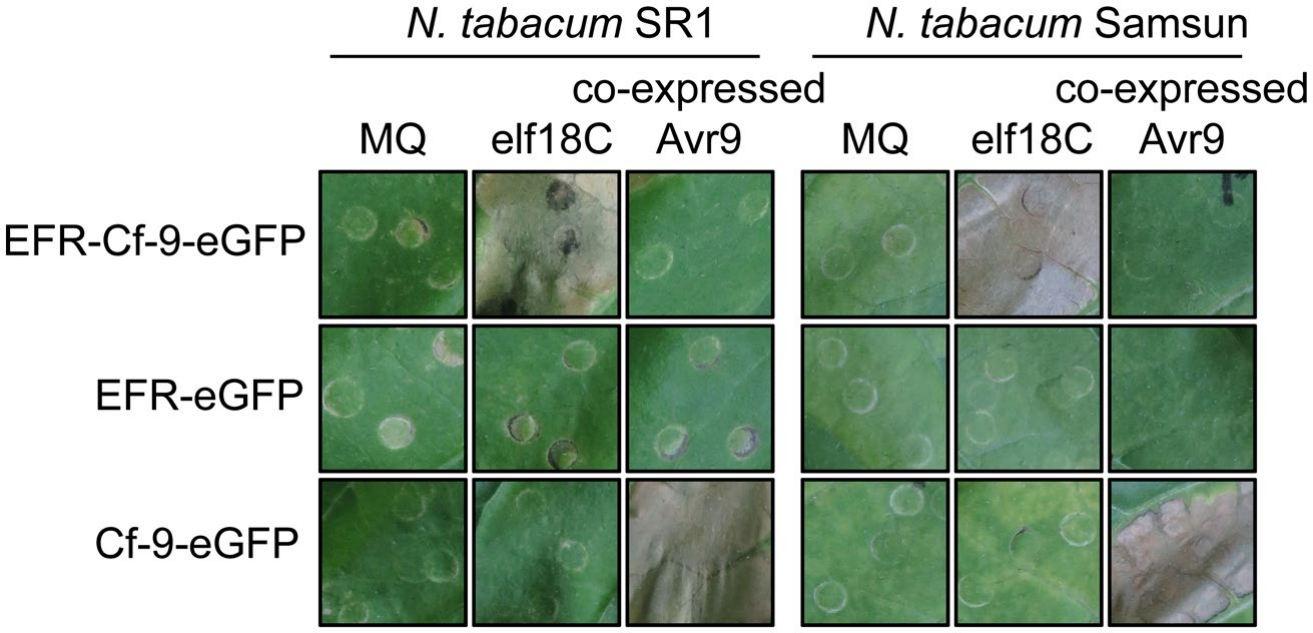
EFR‐Cf‐9 recognizes elf18C and triggers an HR. EFR‐Cf‐9, EFR and Cf‐9, all fused to eGFP, were transiently expressed in wild type (WT) *N. tabacum* SR1 (left) and *N. tabacum* Samsun (right) plants (*n* = 4). After 2 days post agroinfiltration, leaves were treated with Milli‐Q water (MQ) or 100 nM elf18C and pictures were taken after 2 days. As a positive control, eGFP‐tagged EFR‐Cf‐9, EFR and Cf‐9 were also co‐infiltrated with an *Agrobacterium* strain driving expression of Avr9, and pictures were taken at 12 days post‐infiltration (dpi). At these time points, all leaves agroinfiltrated with EFR‐Cf‐9 and subsequently infiltrated with el18C, and all leaves co‐expressing Cf‐9 and Avr9 showed necrosis of at least half of the infiltrated area, whereas none of the other leaves showed any HR‐like symptoms. Experiments were repeated three times with similar results. Representative images are shown.

We previously reported that the ectodomain of EFR (Lys649_EFR_) is functional in an EFR‐WAK1 chimera and perceives elf18, while the ectodomain of FLS2 (Arg806_FLS2_) is functional in an FLS2‐EFR chimera (eJMC in the original article) and perceives flg22 (Brutus *et al*., 2010). Here, we fused the ectodomains of EFR or FLS2 to the endodomain (Trp811_Cf‐9_) of Cf‐9 (Jones *et al*., 1994), to obtain EFR‐Cf‐9 and FLS2‐Cf‐9 chimeras, respectively (Fig. 1). Both chimeras were in turn fused to eGFP and transiently expressed by agroinfiltration in leaves of tobacco plants stably overexpressing *Avr9* (Hammond‐Kosack *et al*., 1998) or transiently co‐expressing Avr9. In parallel, EFR‐eGFP, FLS2‐eGFP and Cf‐9‐eGFP fusions were agroinfiltrated as controls. Leaf areas transiently expressing eGFP‐tagged EFR, EFR‐Cf‐9, FLS2 and FLS2‐Cf‐9 did not exhibit any symptom, while leaves co‐expressing Cf‐9‐eGFP and Avr9, as expected, displayed a clear HR (Figs S1 and S2). These results indicate that the chimeras are not responsive to Avr9 and do not *per se* induce HR‐like symptoms. Next, whether EFR‐Cf‐9 and FLS2‐Cf‐9 are functional and trigger a ligand‐dependent HR‐like response was tested. Upon infiltration with 100 nM elf18C, leaf areas transiently expressing EFR‐Cf‐9‐eGFP showed a strong HR, similar to the Cf‐9‐eGFP/Avr9 control, in two different tobacco cultivars (SR1 and Samsun) (Fig. 2). In contrast, leaf areas transiently expressing FLS2‐Cf‐9‐eGFP did not show visible symptoms upon treatment with 100 µM flg22 (Fig. S2), suggesting that FLS2‐Cf‐9‐eGFP is not functional in tobacco.

Together, these data show that EFR‐Cf‐9 recognizes elf18C to trigger a strong Cf‐9/Avr9‐like HR, indicating that while the ectodomain of EFR retained the ability to perceive the elf18C peptide, its fusion to the endodomain of Cf‐9 resulted in an output similar to that of the native Cf‐9.

### EFR‐Cf‐9 functionality requires SOBIR1 and BAK1

**Figure 3:**
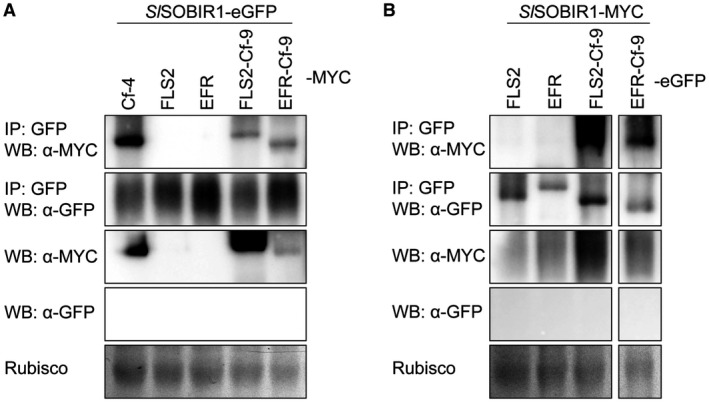
Both FLS2‐Cf‐9 and EFR‐Cf‐9 interact with SOBIR1. (A) eGFP‐tagged tomato (*Sl*) SOBIR1 was transiently co‐expressed with Myc‐tagged Cf‐4, FLS2, EFR, FLS2‐Cf‐9 and EFR‐Cf‐9. (B) Myc‐tagged *Sl*SOBIR1 was transiently co‐expressed with eGFP‐tagged FLS2, EFR, FLS2‐Cf‐9 and EFR‐Cf‐9.Agrobacteria driving expression of the various constructs were infiltrated at a final OD_600 _of 1.0. After 2 days, proteins were extracted and immunoprecipitated (IP) using GFP‐Trap beads. Proteins were detected by Western blot/immunoblot (WB) using α‐GFP and α‐Myc antibodies. Equal loading is indicated by Rubisco band. Experiments were repeated three times and representative images are shown.

It has been reported that the TM domain of SOBIR1 is required for its interaction with Cf‐4 (Bi *et al*., 2016) and that the co‐receptors SOBIR1 and BAK1 are both required for the function of Cf‐4 (Liebrand *et al*., 2013; Postma *et al*., 2016). Cf‐9 shares an identical endodomain with Cf‐4 (Thomas *et al*., 1997) and also interacts with SOBIR1 (Liebrand *et al*., 2013). We therefore investigated whether EFR‐Cf‐9 also interacts with tomato SOBIR1 (henceforth indicated as *Sl*SOBIR1). EFR‐Cf‐9‐Myc was generated and co‐expressed with SISOBIR1‐eGFP in *N. benthamiana*. In parallel, FLS2‐Cf‐9 (as the non‐functionality of this chimera might be due to a possible lack of interaction with SOBIR1), Cf‐4‐eGFP, FLS2‐eGFP and EFR‐eGFP were agroinfiltrated as controls. Immunoprecipitation (IP) of *Sl*SOBIR1 using GFP‐Trap beads demonstrated that Cf‐ was co‐precipitated with *Sl*SOBIR1 (Fig. 3A), which is consistent with our previous finding (Liebrand *et al*., 2013). In addition, FLS2‐Cf‐9 and EFR‐Cf‐9 were also co‐purified with *Sl*SOBIR1, indicating that both chimeras constitutively interact with *Sl*SOBIR1, similar to the Cf proteins (Fig. 3A). In reverse co‐immunoprecipitation assays (co‐IPs), IP of both FLS2‐Cf‐9‐eGFP and EFR‐Cf‐9‐eGFP resulted in the co‐precipitation of *Sl*SOBIR1‐Myc (Fig. 3B). In both cases, the FLS2 and EFR wild‐type proteins did not interact with *Sl*SOBIR1 (Fig. 3A and B), confirming the specificity of SOBIR1 for RLPs (Liebrand *et al*., 2013, 2014).

**Figure 4:**
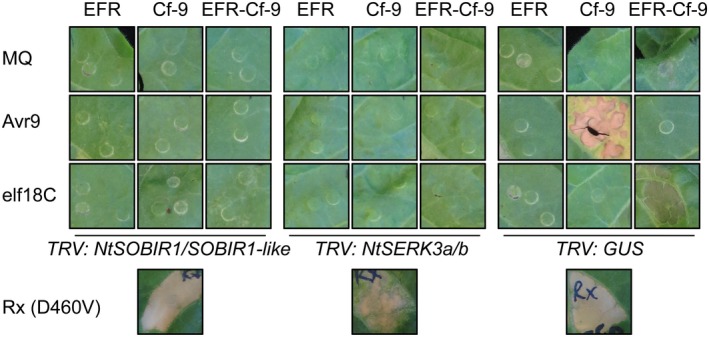
SOBIR1 and BAK1 are required for the EFR‐Cf‐9‐dependent elf18C‐triggered HR. eGFP‐tagged EFR, Cf‐9 and EFR‐Cf‐9 were either transiently expressed with Avr9, or expressed alone, in *N. tabacum *Samsun plants silenced for *NtSOBIR1/SOBIR1‐like*, *NtSERK3a/b* or *GUS *(*n* = 4). *Agrobacteria* driving expression of the constructs were infiltrated at a final OD_600 _of 1.0, except for the positive control Rx (D460V), which was infiltrated at an OD_600 _of 0.1. At 2 days post‐infiltration, leaves were treated with Milli‐Q water (MQ) or 100 nM elf18C. Pictures were taken at 2 days after treatment with elf18C or, in the case of Cf‐9‐Avr9 interaction, at 12 days after co‐infiltration of Cf‐9 and Avr9. Experiments were repeated three times with similar results and representative images are shown.

Next, we investigated whether EFR‐Cf‐9 requires SOBIR1 and/or BAK1 to trigger an HR upon perception of elf18C. Tobacco Samsun plants were used for silencing *NtSOBIR1/SOBIR1‐like* and *NtSERK3a/b*, orthologues of SOBIR1 and BAK1, respectively (Heese *et al*., 2007; Liebrand *et al*., 2013). eGFP‐tagged EFR, Cf‐9 and EFR‐Cf‐9 were transiently co‐expressed with Avr9 or expressed alone in the silenced plants, followed by treatment with Milli‐Q (MQ) water or 100 nM elf18C. Consistent with earlier studies (Liebrand *et al*., 2013; Postma *et al*., 2016), silencing of *NtSOBIR1/SOBIR1‐like* and of *NtSERK3a/b* strongly suppressed the HR triggered by the Cf‐9/Avr9 combination, but did not affect the ability of the plant to mount PCD triggered by Rx(D460V) (Fig. 4), which is a constitutively active form of the *Rx* gene, providing resistance against Potato Virus X (PVX) (Bendahmane *et al*., 2002). The EFR‐Cf‐9‐dependent elf18C‐triggered HR was also severely compromised in the silenced plants, indicating that both *NtSOBIR1/SOBIR1‐like* and *NtSERK3a/b* are required for EFR‐Cf‐9 functionality (Fig. 4). Control plants, infected with Tobacco Rattle Virus carrying the β‐*glucuronidase* (GUS) gene (TRV‐GUS), did not show any defect in the EFR‐Cf‐9‐dependent HR (Fig. 4).

**Figure 5:**
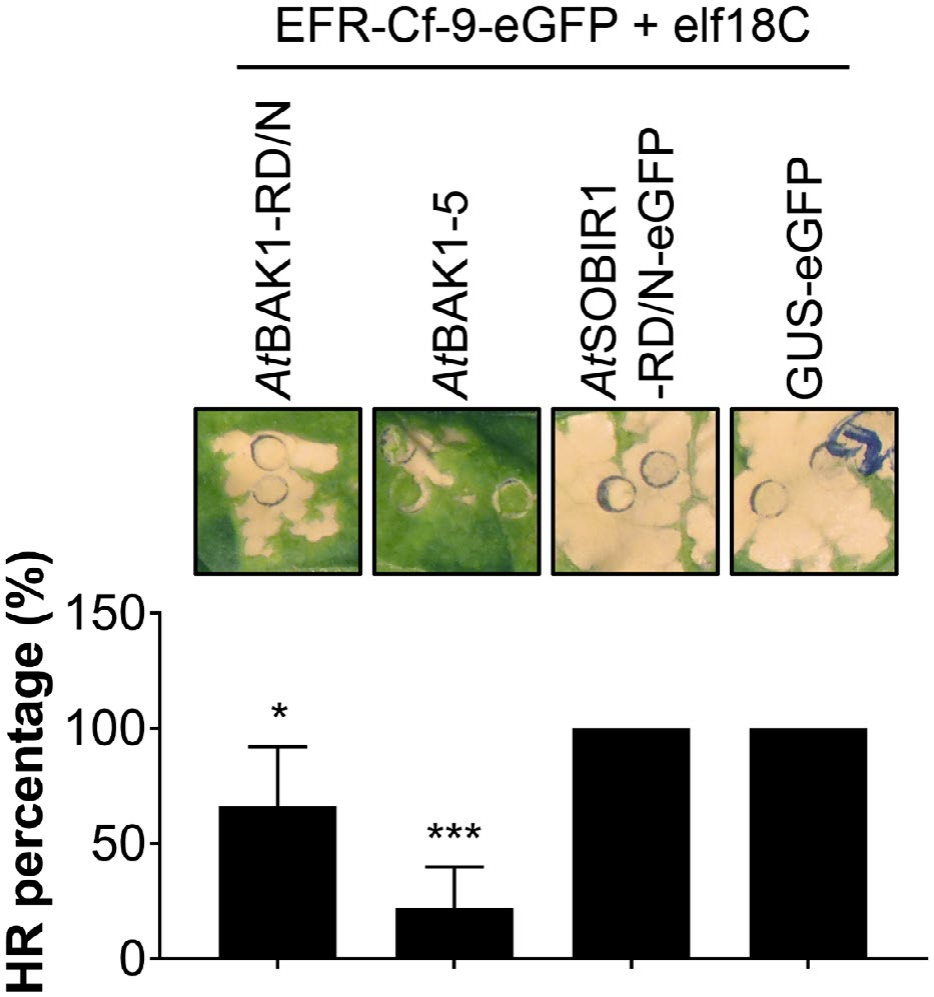
EFR‐Cf‐9‐dependent elf18C‐triggered HR requires kinase activity of BAK1. EFR‐Cf‐9‐eGFP was transiently co‐expressed with the kinase‐dead mutant *At*BAK1‐RD/N, the hypoactive mutant *At*BAK1‐5, the kinase‐dead mutant *At*SOBIR1‐RD/N‐eGFP and GUS‐eGFP at a final OD_600 _of 1.0 (*n* = 5) in *N. tabacum* Samsun plants. At 2 days post‐infiltration, leaves were treated with 100 nM elf18C, and pictures were taken at 2 days after treatment. Bars in the bottom panel show the average percentage of the infiltrated area showing necrosis ± standard deviation (SD) (*n* = 5). The asterisks indicate significant difference with GUS‐GFP, determined by Student's *t‐*test (*P < 0.05, ***P < 0.001). Experiments were repeated three times with similar results, and representative images are shown (top panel).

Kinase activity of both SOBIR1 and BAK1 is required for the function of Cf‐4 (Liebrand *et al*., 2013; Postma *et al*., 2016; Van Der Burgh *et al*., 2018). The *At*BAK1‐5 mutant contains a point mutation in its kinase domain, which causes this protein to have a slightly lower kinase activity than BAK1 itself (Schwessinger *et al*., 2011). BAK1‐5 has been reported to have a dominant‐negative effect on the BAK1‐dependent immune response mediated by FLS2 and EFR (Schwessinger *et al*., 2011). In order to study whether kinase activity of BAK1 is also required for EFR‐Cf‐9 functionality and whether the kinase‐inactive variant *At*SOBIR1‐RD/N (Bi *et al*., 2016) displays a dominant‐negative effect similar to the hypoactive kinase *At*BAK1‐5 and kinase‐dead *At*BAK1‐RD/N mutants (Schwessinger *et al*., 2011), EFR‐Cf‐9‐eGFP was either transiently expressed in combination with *At*BAK1‐RD/N, *A*tBAK1‐5, *At*SOBIR1‐RD/N‐eGFP or GUS‐eGFP, followed by treatment with 100 nM elf18C. The EFR‐Cf‐9‐dependent elf18C‐triggered HR was not affected by GUS‐eGFP, but was reduced by *At*BAK1‐RD/N and even more compromised by *At*BAK1‐5 (Fig. 5), indicating that both BAK1 mutants have a dominant‐negative effect on the EFR‐Cf‐9‐triggered HR, possibly because the overexpressed BAK1 mutants compete away the endogenous *Nt*SERK3a/b from the activated EFR‐Cf‐9‐containing signalling complex. We conclude that the EFR‐Cf‐9‐dependent elf18C‐triggered HR depends on the kinase activity of BAK1. Unlike *At*BAK1‐RD/N and *At*BAK1‐5, *At*SOBIR1‐RD/N‐eGFP did not affect the HR (Fig. 5). Considering that RLPs constitutively interact with SOBIR1, whereas the recruitment of BAK1 to the signalling complex is ligand‐dependent, it is possible that transiently expressed *At*SOBIR1‐RD/N‐eGFP may fail to displace the endogenous *Nt*SOBIR1/SOBIR1‐like protein that is bound to EFR‐Cf‐9.

Taken together, these data indicate that both SOBIR1 and BAK1 are required for the EFR‐Cf‐9‐dependent elf18C‐triggered HR, and that BAK1 has to be kinase‐active.

### *EFR‐Cf‐9*‐transgenic tobacco plants are differentially responsive to elf18 variants

**Figure 6:**
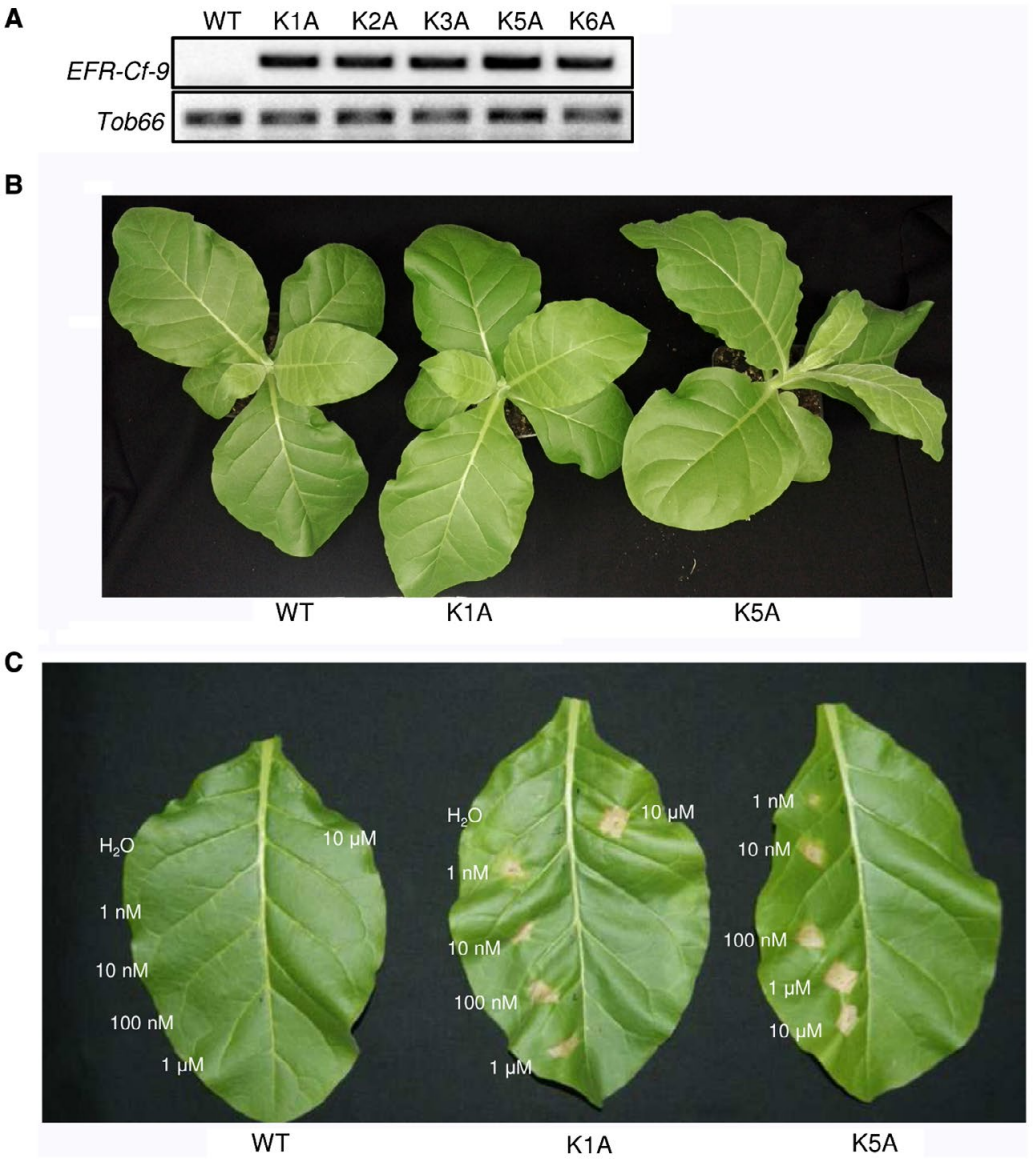
*EFR‐Cf‐9*‐transgenic plants do not show morphological changes and recognize elf18C to trigger HR. (A) Expression of *EFR‐Cf‐9* in leaves of wild‐type (WT) plants and five primary transformants (K1A, K2A, K3A, K5A and K6A) was evaluated by Reverse Transcription‐Polymerase Chain Reaction (RT‐PCR), using *Tob66* as a control. (B) Picture of representative 4‐week‐old WT and transgenic K1A and K5A plants. (C) Fully expanded leaves of 4‐week‐old WT and *EFR‐Cf‐9*‐transgenic plants (K1A and K5A) (*n* > 3) were infiltrated with elf18C at the indicated concentrations. Pictures were taken at 5 days post‐infiltration. All leaves from transgenic plants showed necrosis of at least 50% of the area infiltrated with elf18C, whereas those infiltrated with water and all WT leaves failed to display HR‐like symptoms. Experiments were repeated three times and representative images are shown.

An untagged version of *EFR‐Cf‐9* was stably expressed in tobacco SR1 plants. Fourteen primary transformants were obtained, of which five had detectable expression of *EFR‐Cf‐9* (Fig. 6A) and were therefore propagated. Two independent lines (K1A and K5A) showing a 3:1 segregation ratio for kanamycin resistance, and therefore likely carrying an insertion in a single locus, were selected for further characterization. Transgenic plants were morphologically identical to the parental plants (Fig. 6B). To verify that the chimera was properly expressed and localized, trypsin digestion of microsomal leaf proteins from wild type (WT) and K1A plants was performed, followed by Liquid chromatography‐tandem mass spectrometry (LC‐MS/MS). Six peptides corresponding to the ectodomain of EFR (coverage of 15.49; score of 16.31) were found only in extracts from the transgenic plants (Fig. S3), confirming that EFR‐Cf‐9 is expressed and is likely membrane‐localized. No peptide corresponding to the chimera was found in WT extracts.

Subsequently, the functionality of EFR‐Cf‐9 in K1A and K5A plants was assessed. Only leaves from both transgenic lines mounted an HR upon elf18C treatment, at concentrations ranging from 1 nM to 10 μM (Fig. 6C). Responsiveness to different elf18 variants was also tested in line K1A, revealing that an HR was triggered also by elf18B and elf18G, though to a lesser extent when compared with elf18C (Fig. S4). This suggests that, although the EFR ectodomain was fused to the endodomain of Cf‐9, the structure of the ligand still determines the output. To obtain a more quantitative evaluation of the response mediated by EFR‐Cf‐9, and to assess whether the presence of EFR‐Cf‐9 might cause an elevated basal defence response, the expression of the marker genes *Avr9/Cf‐9 RAPIDLY ELICITED‐132* (*ACRE‐132*) (Durrant *et al*., 2000) and *HAIRPIN INDUCED 1* (*HIN1*) (Gopalan *et al*., 1996) was analysed. Transcript levels of both genes were similar in MQ water‐treated WT and K1A plants, indicating that *EFR‐Cf‐9* does not affect basal defence (Fig. S5). In addition, as expected, WT plants did not respond to any of the elf18 variants tested, whereas they showed a significantly increased expression of *ACRE‐132* and *HIN1* after flg22 elicitation (Fig. S5). In contrast, treatment of K1A plants with elf18C induced a significant increase in transcript levels for *ACRE‐132* and *HIN1*, which were even higher than in plants treated with flg22 (Fig. S5). Moreover, treatment with elf18B and elf18G also resulted in increased transcript levels for *ACRE‐132* and *HIN1*, but to a lesser extent than for elf18C (Fig. S5), which supports our finding that elf18C triggers a stronger HR than the elf18 variants (Fig. S4). On the other hand, WT and K1A plants displayed similar expression levels of both genes in response to flg22 (Fig. S5), indicating that *EFR‐Cf‐9* does not affect the endogenous *Nt*FLS2‐mediated response to flg22.

Together, these data indicate that transgenic tobacco plants expressing *EFR‐Cf‐*9 are not altered in their basal defence and in their responsiveness to flg22. Moreover, the *EFR‐Cf‐9*‐transgenic plants respond more efficiently to elf18C than to the different elf18 variants.

### Transgenic tobacco plants expressing *EFR‐Cf‐9* show enhanced resistance to (hemi)biotrophic bacterial pathogens

**Figure 7:**
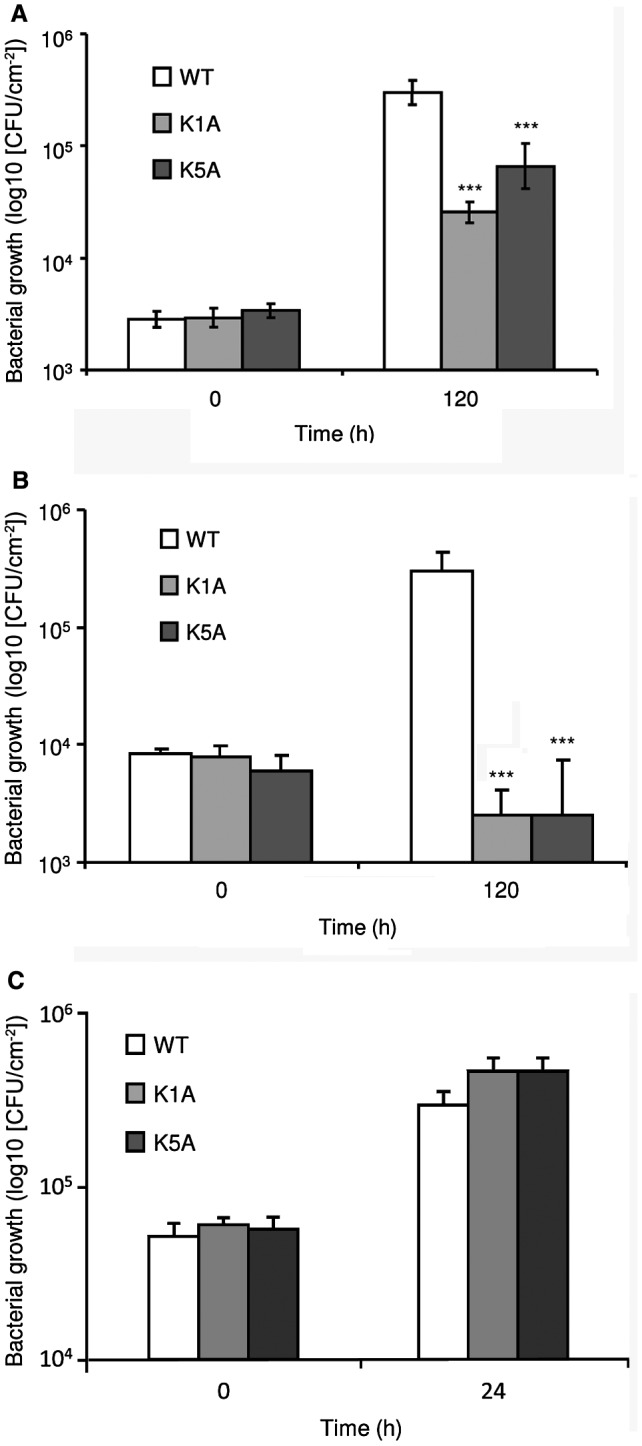
*EFR‐Cf‐9*‐transgenic plants show altered resistance to bacterial pathogens. Fully expanded leaves of 4‐week‐old untransformed plants wild type (WT) and *EFR‐Cf‐9*‐transgenic plants (K1A and K5A) were inoculated with *Pseudomonas amygdali* pv. *tabaci *11528 (A) or *Pseudomonas syringae* pv *tomato* DC3000 (B) at an initial density of OD_600_ = 0.002 (approximately 10^4^ CFU/cm^2^), or *Pectobacterium carotovorum* (C) at OD_600_ = 0.02 (approximately 10^5^ CFU/cm^2^). Bacterial colonization was determined at the indicated time points (*n* > 3 for each time point). Asterisks indicate statistically significant differences between WT and transgenic lines, according to Student's *t*‐test (***P < 0.0001).

**Figure 8:**
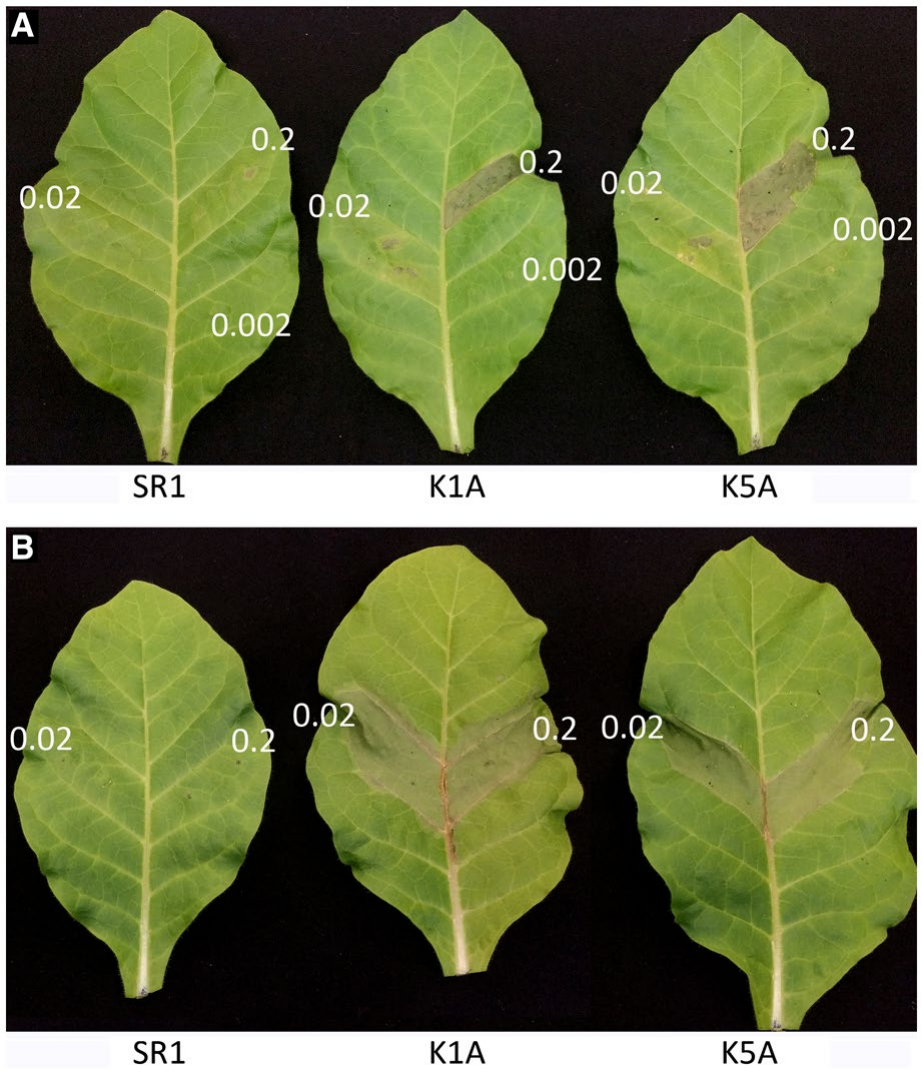
*EFR‐Cf‐9 *transgenic plants show hypersensitive response (HR) to high doses of bacterial pathogens. Leaf sectors of fully expanded leaves of 4‐week‐old untransformed plants wild type (WT) and EFR‐Cf‐9 K1A and K5A transgenic plants were inoculated with *Pseudomonas amygdali* pv. *tabaci *11528 (A) at OD_600_ = 0.002, 0.02 or 0.2 (about 10^4^, 10^5^ or 10^6^ CFU/cm^2^, respectively), or with *Pectobacterium carotovorum* (B) at OD_600 _= 0.02 or 0.2, corresponding to a bacterial density of about 10^5^ or 10^6^ CFU/cm^2^, respectively (*n* > 3 for each treatment). Pictures were taken 48 h (A) or 24 h (B) after inoculation. Experiments were repeated three times with similar results, and representative images are shown.

To study whether the EFR‐Cf‐9‐mediated immune response eventually leads to resistance against bacterial pathogens, WT and transgenic K1A and K5A plants were inoculated with the (hemi)biotrophic bacterial pathogens *Pta* 11528 and *Pst* DC3000 at a dose of OD_600_ = 0.002 (corresponding to 10^4^ CFU/cm^2^). Compared to WT, a clear reduction in bacterial colonization was found in both transgenic lines inoculated with *Pta* 1152 and, to an even greater extent, *Pst* DC3000 (Fig. 7A and B). To verify that EFR‐Cf‐9 indeed triggers an HR not only in response to the purified elf18 peptides, but also to bacterial infection, we inoculated different doses (OD_600_ = 0.002, 0.02 and 0.2) of *Pta* 11528 in WT, K1A and K5A plants. Within 48 h, leaf sectors of the transgenic plants inoculated with the highest doses of bacteria (OD_600_ = 0.02 and 0.2) displayed HR‐like symptoms, whereas WT plants did not show any symptoms with all doses of bacteria (Fig. 8A).

We also tested the susceptibility to the necrotrophic bacterium *Pectobacterium carotovorum* subsp*. carotovorum* strain DSMZ 30169, which is the causal agent of bacterial soft rot. Inoculation with *P. carotovorum* is able to induce an EFR‐dependent response in Arabidopsis, indicating the presence of an elf18‐like MAMP (Lacombe *et al*., 2010). Leaves inoculated with *P. carotovorum* at a density of OD_600_ = 0.02 (corresponding to about 10^5^ CFU/cm^2^) showed a ten‐fold increase in bacterial count at 24 h, with no significant differences between WT and the transgenic lines (Fig. 7C). Notably, also in this case we observed a rapid and strong HR after inoculation with bacteria in K1A and K5A but not in WT plants (Fig. 8B). This suggests that EF‐Tu of *P. carotovorum* is recognized by EFR‐Cf‐9 and triggers an HR that is not able to restrict colonization by this pathogen.

The fungal pathogen *Botrytis cinerea* does not produce an elf18‐like MAMP and its colonization in Arabidopsis is not affected by the lack of EFR (Brutus *et al*., 2010). Consistently, when *B. cinerea *was inoculated onto WT and transgenic K1A and K5A leaves, no differences in disease severity was observed (Fig. S6), indicating that the ectodomain of EFR‐Cf‐9 does not recognize any MAMP from *B. cinerea *and that basal defence is not enhanced by the presence of the chimera.

Taken together, these data indicate that pathogen‐derived elf18‐like MAMPs activate EFR‐Cf‐9‐dependent immunity resulting in an HR, which restricts (hemi)biotrophic bacteria colonization, but does not compromise colonization by necrotrophic bacteria or fungi.

## Discussion

### The chimera EFR‐Cf‐9 recognizes elf18 to trigger a strong immune response

Interfamily transfer of PRRs is a promising strategy to confer broad‐spectrum resistance to pathogens, and it has been successfully employed with EFR (Lacombe *et al*., 2010; Schwessinger *et al*., 2015), Xa21 (Afroz *et al*., 2011; Holton *et al*., 2015; Mendes *et al*., 2010; Tripathi *et al*., 2014) and Ve1 (Song *et al*., 2017). Engineering chimeric receptors that combine the properties of two separate PRRs is another effective strategy to improve host resistance. Current research on chimeric PRRs has revealed that the ectodomain determines ligand specificity, while the endodomain determines output intensity (Brutus *et al*., 2010; He *et al*., 2000; Holton *et al*., 2015; Kishimoto *et al*., 2010; Kouzai *et al*., 2013; Schwessinger *et al*., 2015). Here we generated the chimera EFR‐Cf‐9 (Fig. 1), combining the ectodomain of EFR, which provides broad‐spectrum recognition of bacterial pathogens, and the endodomain of Cf‐9, which induces a strong HR‐associated immune response. We demonstrated that, when expressed in tobacco plants, EFR‐Cf‐9 recognizes elf18 leading to an HR (Fig. 2). We also showed that EFR‐Cf‐9 interacts with SOBIR1, similar to the Cf‐9 protein itself (Fig. 3), and requires both SOBIR1 and BAK1 for its function (Fig. 4). In addition, EFR‐Cf‐9 also recognizes pathogen‐derived elf18 or elf18‐like MAMPs to activate immunity (Fig. 7) and triggers HR during bacterial infection (Fig. 8). Moreover, EFR‐Cf‐9 retains the recognition feature of EFR as elf18C, compared to elf18 variants with lower EFR‐eliciting activity, triggers a stronger response, (Figs S4 and S5).

In contrast to EFR‐Cf‐9, FLS2‐Cf‐9 failed to trigger an HR upon treatment with flg22 (Fig. S2), although FLS2‐Cf‐9 also interacts with SOBIR1 in *N. benthamiana* (Fig. 3). Although the FLS2 ectodomain is functional in FLS2‐EFR (Brutus *et al*., 2010) and the Cf‐9 endodomain is functional in EFR‐Cf‐9 (Fig. 2), it is still possible that the selected point of junction between the ectodomain of FLS2 and the endodomain of Cf‐9 might not be optimal for functionality of this specific chimera.

### Early transduction events mediated by the EFR‐Cf‐9 chimera are similar to those employed by Cf‐9

Although interfamily PRR transfer and expression of chimeric PRRs can provide broad resistance to pathogens (Afroz *et al*., 2011; Holton *et al*., 2015; Lacombe *et al*., 2010; Schoonbeek *et al*., 2015), the downstream signalling components employed by these receptors are barely known. Indeed, the signalling partners downstream of the perception event are not always conserved in the recipient plant. For example, *Os*SERK2, which is phylogenetically closely related to *At*SERK1 and *At*SERK2, is required for the functionality of transgenically expressed EFR in resistance to *Xanthomonas* *oryzae *pv. *oryzae *(*Xoo*) in rice (Chen *et al*., 2014). However, in Arabidopsis *At*SERK1 and *At*SERK2 are not required for EFR function (Roux *et al*., 2011), indicating that EFR utilizes different SERKs in *Arabidopsis* and rice. In addition, specific downstream signalling components may act in an opposite manner in different species. For instance, the Xa21‐binding (XB) protein *Os*XB24 is an ATPase that negatively affects both Xa21‐ and EFR‐mediated immunity in rice (Chen *et al*., 2010; Schwessinger *et al*., 2015), while its orthologue plays a positive role in EFR‐mediated immunity in *Arabidopsis *(Holton *et al*., 2015). Thus, the components required for functionality of a transferred PRR are not easily predictable.

Ectopic expression of chimeric receptors allows to address the requirement of specific protein domains for physical and functional interaction with partners participating in downstream signalling. For example, both EFR‐Xa21 and native Xa21 form a constitutive complex with *Os*SERK2, indicating that *Os*SERK2 interaction with Xa21 does not specifically require the Xa21 ectodomain. In Arabidopsis, both Xa21 and EFR‐Xa21 interact with BAK1 in a ligand‐dependent manner (Schwessinger *et al*., 2015). In this study, we show that EFR‐Cf‐9 constitutively interacts with SOBIR1 and requires SOBIR1 for its function (Figs 3 and 4). EFR‐Cf‐9 is anticipated to also form a complex with BAK1 upon elf18 treatment, as the chimera also requires kinase‐active BAK1 for its function (Fig. 5). It should be noted that *At*BAK1‐5 had a stronger effect than *At*BAK1‐RD/N in suppressing EFR‐Cf‐9‐mediated HR (Fig. 5). The interaction between *At*BAK1 and EFR is kinase activity‐independent and *At*BAK1‐5 has a higher affinity to EFR than WT *At*BAK1 (Schwessinger *et al*., 2011), explaining why *At*BAK1‐5 is more efficient than *At*BAK1‐RD/N in suppressing EFR‐Cf‐9 activity.

SOBIR1 constitutively interacts with Cf‐4, which shares an identical endodomain with Cf‐9 (Liebrand *et al*., 2013; Thomas *et al*., 1997), whereas it does not interact with the RLKs FLS2 and EFR and is not required for RLK‐mediated immunity (Gust and Felix, 2014). The external juxtamembrane region of EFR‐Cf‐9 does not carry the typical stretch of acidic amino acids that are thought to play a role in the interaction of Cf‐9 with SOBIR1 (Bi *et al*., 2016; Gust and Felix, 2014). However, EFR‐Cf‐9 does interact with SOBIR1, suggesting that the TM of Cf‐9, carrying an extensive GxxxGxxxGxxxG dimerization motif, is sufficient for interaction with SOBIR1 (Bi *et al*., 2016). The requirement of the GxxxG motif for the interaction between SOBIR1 and EFR‐Cf‐9 will be investigated by performing site‐directed mutagenesis of this motif in future studies. Together, this indicates that the endodomain, and in particular the TM, of Cf‐9 provides the chimera EFR‐Cf‐9 with the features necessary for Cf‐9/Cf‐4 signalling, enabling constitutive interaction with SOBIR1 and ligand‐dependent BAK1 recruitment to the EFR‐Cf‐9/SOBIR1 complex, eventually mounting an HR.

### Transgenic expression of *EFR‐Cf‐9* affects resistance against bacterial pathogens

In tomato, Cf proteins mediate a strong resistance to the *C. fulvum* strains carrying the proper avirulence genes (Joosten *et al*., 1997; Stergiopoulos and de Wit, 2009). It is widely accepted that this resistance is largely based on the HR resulting from the activation of the Cf protein, which leads to localized cell death that restricts pathogen spread. However, the strong resistance mediated by *Cf* genes has a narrow specificity, and is easily overcome by mutations in the corresponding *Avr* genes of the pathogen. On the other hand, PRR‐mediated immunity triggered upon recognition of MAMPs is effective against a wide range of microbes but is weaker than that of Cf proteins. Hence, the generation of a chimera containing the ectodomain of a PRR and the endodomain of a Cf protein might combine the beneficial features of PRRs and Cf proteins, and enable the chimera to recognize MAMPs and mount an HR, leading to a strong immunity to a wide range of microbes.

EFR‐Cf‐9 recognizes elf18 to mount an HR (Fig. 2), suggesting that it might also recognize pathogen‐derived elf18 to activate immunity. Indeed, transgenic tobacco plants expressing *EFR‐Cf‐9* showed a significantly reduced susceptibility to two (hemi)biotrophic bacterial pathogens, *Pta* 11528 and *Pst* DC3000 (Fig. 7A and B). In addition, inoculation of a high dose of *Pta* 11528 causes HR in the transgenic plants (Fig. 8A), indicating that EFR‐Cf‐9 recognizes both the purified elf18 peptide and the pathogen‐derived EF‐Tu, and that this recognition effectively restricts bacterial growth. However, compared to *Pst* DC3000, EFR‐Cf‐9‐mediated resistance to *Pta* 11528 is less efficient (Fig. 7A and B), suggesting that *Pta* 11528 might partially suppress the EFR‐Cf‐9‐mediated immune response, as *Pta* 11528 has been shown to suppress salicylic acid‐mediated defence responses (Lee *et al*., 2013).

Inoculation of the necrotrophic bacterium *P. carotovorum* in the *EFR‐Cf‐9*‐transgenic plants also resulted in HR‐like symptoms (Fig. 8B), since *P. carotovorum* carries a form of EF‐Tu that is able to activate EFR (Lacombe *et al*., 2010). However, no significant difference in *P. carotovorum *growth between WT and transgenic plants was found (Fig. 7C).

## Conclusion

This work indicates that the EFR‐Cf‐9 chimera is functional in tobacco plants, since it recognizes elf18 to trigger an HR, which fits our current view on the ectodomain of a PRR determining the ligand specificity, and the endodomain determining the output intensity. Moreover, EFR‐Cf‐9 constitutively interacts with SOBIR1, and the EFR‐Cf‐9‐mediated HR triggered by elf18 is dependent on SOBIR1 and BAK1, which is reminiscent of our working model for Cf‐4/Cf‐9 and indicates that the endodomain of Cf‐9 confers EFR‐Cf‐9 the repertoire of Cf‐9/Cf‐4‐like signalling. In addition, transgenic tobacco plants expressing *EFR‐Cf‐9* do not show altered basal defence but are resistant to (hemi)biotrophic bacteria, suggesting a potential biotechnological use of this chimeric receptor to improve crop disease resistance. Furthermore, our construction of the EFR‐Cf‐9 chimera reveals that it is possible to customize crop immunity by the generation of chimeric receptors containing features of a varying ligand recognition range and immune response intensity.

## Experimental procedures

### Plant materials and growth conditions

*N. benthamiana*, *N. tabacum *Samsun, *N. tabacum* SR1, *Avr9*‐transgenic *N. tabacum* SR1 (Hammond‐Kosack *et al*., 1998), and *EFR‐Cf‐9‐*transgenic *N. tabacum* SR1 plants were grown under 16 h of light at 25 °C, and 8 h of darkness at 21 °C in climate chambers with a relative humidity of 75%.

### Constructs

The DNA fragments representing the coding sequence (CDS) of the ectodomain of EFR (Lys649_EFR_) and FLS2 (Arg806_FLS2_) (Brutus *et al*., 2010) were fused to the CDS of the TM and cytoplasmic domain of Cf‐9 by splicing overlapping extension polymerase chain reaction (SOE‐PCR) (Higuchi *et al*., 1988) to generate DNA fragments encoding EFR‐Cf‐9 and FLS2‐Cf‐9, respectively, and cloned into the binary vector pBI121 under the control of the 35S promoter, or, by Gateway cloning, the fragments were inserted into pK7FWG2.0 to generate 35S:EFR‐Cf‐9‐eGFP and 35S:FLS2‐Cf‐9‐eGFP, and inserted into pGWB20 to generate 35S:EFR‐Cf‐9‐10xMyc and 35S:FLS2‐Cf‐9‐10xMyc.

Construction of *Sl*SOBIR1‐eGFP, *Sl*SOBIR1‐Myc, Cf‐4‐Myc, FLS2‐Myc and EFR‐Myc was reported earlier (Liebrand *et al*., 2013). The construction of *At*SOBIR1‐RD/N‐eGFP was reported by Bi *et al*. (2016). *At*BAK1‐5 (SOL5114) and *At*BAK1‐RD/N (SOL5106) were previously reported (Schwessinger *et al*., 2011). Binary vectors carrying 35S:Cf‐9 and 35S:Avr9 in pMOG800 have been described (Van der Hoorn *et al*., 2000). All plasmids were transferred into *A. tumefaciens *C58C1, carrying the helper plasmid pCH32.

### Generation of transgenic tobacco plants

Transformation of tobacco plants was performed according to established methods (Horsch *et al*., 1988). Briefly, *A. tumefaciens* carrying the 35S:EFR‐Cf‐9 plasmid were suspended in an infection medium composed as follows: Murashige‐Skoog (MS) basal medium, 3% w/v sucrose, 200 µM acetosyringone, 0.001% v/v Silwet. Leaf discs of 1 cm of diameter from 4‐week‐old tobacco SR1 leaves were incubated in the infection medium for 30 min at 25 ºC. Leaf discs were then placed on co‐culturing MS medium containing Gamborg's vitamin mix, 1 mg/L 6‐BAP, 0.1 mg/L NAA, 200 µM acetosyringone at 25 °C in the dark for 2 days. Infected explants were then transferred to fresh solid MS medium supplemented with vitamin mix, 1 mg/L 6‐BAP, 0.1 mg/L NAA, 200 mg/L timentin, 200 mg/L cefotaxime and 200 µg/mL kanamycin at 25 °C in the light for 30 days for shoot regeneration. Regenerated shoots were transferred to fresh solid MS medium supplemented with vitamin mix, 0.1 mg/L NAA, 200 mg/L timentin, 200 mg/L cefotaxime and 200 mg/L kanamycin at 25 °C in the light for 30 days for root regeneration. From about 1200 co‐cultivated leaf explants, 14 primary transformants were obtained and transferred to soil for propagation. Five transformants had detectable transgene expression and were propagated; of these, two (K1A and K5A) showed a 3:1 segregation ratio for kanamycin resistance, and were therefore selected for further characterization.

### Hypersensitive response assays

The elf18 (elf18D) and flg22 peptides were synthesized by EZBiolab (Carmel, IN, USA). The other elf18 peptides were kind gifts of Cyril Zipfel (The Sainsbury Laboratory, Cambridge, UK). For HR assays in *N. tabacum* SR1 and *N. tabacum* Samsun plants, fully expanded leaves were infiltrated with *Agrobacterium* suspensions at an OD_600 _of 1.0, except for Rx (D460V), which was infiltrated with an OD_600 _of 0.1 (Liebrand *et al*., 2013; Postma *et al*., 2016). MQ water and elf18 and/or flg22 peptides were infiltrated at 2  days post‐agroinfiltration (dpi) at the indicated concentrations. Leaves were examined for development of an HR between 2 dpi and 12 dpi. For each treatment, at least three leaves, taken from separate plants, were agroinfiltrated.

### Virus‐induced gene silencing (VIGS)

VIGS plasmids pTRV1‐RNA1, pTRV2‐PDS, pTRV2‐GUS, pTRV2‐*Sl*SOBIR1/SOBIR1‐like and pTRV2‐*Sl*SERK3a/b were described before (Liebrand *et al*., 2013; Postma *et al*., 2016). In brief, 2‐week‐old *N. tabacum* Samsun plants were subjected to VIGS, for which *Agrobacterium* cultures harbouring a pTRV2 plasmid were mixed with pTRV1‐RNA1 at a final OD_600 _of 0.8. After about three weeks, fully expanded leaves were used for HR assays.

### Co‐immunoprecipitation and immunoblot assays

Co‐IPs were performed as described previously (Liebrand *et al*., 2012). The following antibodies were used: α‐GFP‐HRP (130‐091‐833, MACS antibodies, Bergisch Gladbach, Germany), α‐cMYC (cMYC9E10, sc‐40, Santa Cruz Biotechnology, Heidelberg, Germany), with α‐Mouse‐HRP (Amersham, GE Healthcare, Eindhoven, Netherlands) as a secondary antibody.

### Pathogenicity assays

*P. amygdali *pv. *tabaci *(*Pta*) 11528 and *P. syringae* pv. *tomato* (*Pst*) DC3000 were grown in 5 mL of low salt Bertani‐Luria (LSBL) medium at 28 °C for 24 h at 200 rpm until OD_600_ was 1.0. Bacteria were centrifuged for 10 min at 2500 × *g*, the supernatant was discarded and bacteria were rinsed twice with sterile water. Bacteria were suspended in an infiltration medium containing 10 mM MgCl_2_ at the indicated doses.

*P. carotovorum* subsp*. carotovorum* strain DSMZ 30169 was obtained from DSMZ GmbH (Germany). Bacteria were grown in liquid Luria‐Bertani (LB) broth at 28 °C for 16 h, centrifuged at 8000 × *g *for 10 min, and washed with sterile water. Bacteria were then suspended in 50 mM potassium phosphate buffer, pH 7.0 and inoculated at the indicated doses.

Bacteria were syringe‐infiltrated into leaf sectors of 4‐week‐old tobacco plants (six sectors per leaf, three leaves per genotype). Inoculated plants were returned to greenhouse and bacterial count was determined after 0 h and 120 h (for *Pta* 11528 and *Pst* DC3000) or 24 h (for *P. carotovorum*). Briefly, inoculated leaf sectors were sterilized for 1 min in EtOH 70%, washed for 1 min in sterile water, and leaf discs (diameter = 0.4 mm) were cut from each sector. Discs were ground with a pestle in 100 μL of sterile water, then additional 900 μL of water were added; samples were vortexed, and serial dilutions were plated on LB solid medium. Plates were incubated at 28 ºC for two days, and colonies were counted for each dilution.

Detached tobacco leaves were inoculated with *B. cinerea* strain SF1, isolated from cabbage (Ferrari *et al.,* 2003), as previously described (Manfredini *et al*., 2005).

### Expression analysis of the *EFR‐Cf‐9 *chimera and the defence‐related genes *HIN1* and *ACRE‐132*

For analysis of the expression of *EFR‐Cf‐9*, total RNA was extracted from leaf sectors of 4‐week‐old plants with Tri‐reagent (Sigma) and treated with Turbo‐DNase I (Ambion). First‐strand cDNA was synthesized using ImProm‐II Reverse Transcriptase (Promega, Madison, WI, USA). Expression of *EFR‐Cf‐9* was evaluated by PCR using specific primers (5′‐ CAAATTCCATCCCTCGCTTA‐3′ and 5′‐TCTTTTCTTGTGCTTTTTCATTTTC‐3′). The tobacco actin gene *Tob66* (accession n. U60491) was amplified using the following primers: 5′‐CTGCCATGTATGTTGCTATT‐3′ and 5′‐AGTCTCCAACTCTTGCTCAT‐3′.

For quantitative analysis of *HIN1* and *ACRE‐132* expression, leaf sectors from 4‐week‐old plants were infiltrated with water or elicitors at the concentration of 100 nM. After 48 h, infiltrated leaf sectors were collected from three separate plants for each genotype. Total RNA was extracted using NucleoZol reagent (MACHEREY‐NAGEL GmbH, Düren, Germany), treated with RQ1 DNase (Promega), and first‐strand cDNA was synthesized using ImProm‐II Reverse Transcriptase (Promega). Quantitative Reverse Transcription‐Polymerase Chain Reaction (qRT‐PCR) analysis was performed using a CFX96 Real‐Time System (Bio‐Rad), using the GoTaq Real‐Time PCR System (Promega). Three technical replicates were performed for each sample, and data were analysed with LinRegPCR, developed at Amsterdam University Medical Centers (AMC) (Ruijter *et al*., 2009). Expression levels of each gene, relative to *EF1a*, were determined as previously described (Ferrari *et al*., 2006), and expressed in arbitrary units. Primer pairs were the following: *HIN1*, 5′‐CTGCAACCCATGTAGCTGTC‐3′ and 5′‐TGTGGTGGACAAATCGAACT‐3′; *ACRE132*, 5′‐GCTGGCGGTTATCAAGAAGT‐3′ and 5′‐TGAAACCCATGATTGCATTT‐3′. *EF1a*, 5′‐GCTCCCACTTCAGGATGTTT‐3′ and 5′‐CCAACATTGTCACCAGGAAG‐3′.

### Mass spectrometry

Membrane‐enriched protein fractions from one leaf of a WT SR1 plant and of a transgenic K1A plant were extracted as previously described (Mattei *et al*., 2016). Proteins were separated by SDS‐PAGE, and lanes were cut into ten slices, that were subjected to in‐gel trypsin digestion. Peptides were analysed by LTQ liquid chromatography (LC) Orbitrap MS/MS, and protein identification was performed by MaxQuant platform and Proteome Discoverer as previously described (Mattei *et al*., 2016).

## Supporting information


**Fig. S1** EFR‐Cf‐9 does not trigger an HR in tobacco plants expressing *Avr*9. eGFP‐tagged EFR, Cf‐9 and EFR‐Cf‐9 were transiently expressed in *Avr*9‐transgenic *N. tabacum* SR1 plants at a final OD_600_ of 1.0. For each construct, at least three leaves, taken from separate plants, were agro‐infiltrated. Pictures were taken at three days post‐infiltration (dpi). The infiltrated areas are indicated by white dashed lines. Under these conditions, all leaves agroinfiltrated with Cf‐9‐eGFP, and none of the other samples, showed necrosis of at least half of the infiltrated area. This experiment was repeated three times with similar results. Representative images are shown.

**Fig. S2** FLS2‐Cf‐9 does not trigger an HR in tobacco plants upon treatment with flg22. eGFP‐tagged FLS2, Cf‐9 and FLS2‐Cf‐9 were either expressed alone (first and third column) or transiently co‐expressed with Avr9 (second column), or, followed by treatment with Milli‐Q (MQ) water (first column) or 100 μM flg22 (third column) after two days. Agrobacteria driving expression of the various constructs were infiltrated at a final OD_600_ of 1.0. Pictures were taken at four days post‐infiltration (dpi) with the elicitor or at seven days of co‐expression with Avr9. The infiltrated areas are indicated by white dashed lines. For each treatment, at least three leaves per construct, taken from separate plants, were infiltrated. Under these conditions, all leaves co‐expressing Avr9 and Cf‐9‐eGFP showed necrosis of at least half of the infiltrated area, whereas none of the other samples showed HR‐like symptoms. This experiment was repeated three times with similar results. Representative images are shown.

**Fig. S3** Identification of EFR Cf 9 in microsomal fractions of transgenic tobacco plants. Amino acid sequence of the EFR Cf 9 chimeric receptor. The five peptides identified by mass spectrometry in microsomal protein fractions extracted from transgenic *EFR‐Cf‐9* expressing K1A leaves are underlined. SP, signal peptide; LRR, leucine rich repeat; eJM, external juxtamembrane; TM, transmembrane; iJM, internal juxtamembrane.

**Fig. S4** EFR Cf 9 transgenic plants recognize different elf18 variants. Fully expanded leaves of 4‐week‐old untransformed wild type (WT) and transgenic tobacco plants expressing EFR Cf 9 (K1A) plants were treated with Milli‐Q (MQ) water or with flg22, elf18C, elf18B or elf18G at a concentration of 100 nM. For each peptide, at least four leaves per genotype, taken from independent plants, were infiltrated. Pictures were taken at 40 h post‐infiltration. The infiltrated area is indicated by the white dashed line. At this time point, all leaves from K1A plants infiltrated with el18C, elf18B or elf18C showed necrosis of at least half of the infiltrated area, whereas those infiltrated with flg22 or water, and all leaves from WT plants, failed to display any HR‐like symptoms. This experiment was repeated three times with similar results, and representative images are shown.

**Fig. S5** Defence‐related genes *ACRE‐132* and *HIN1* are differentially up regulated in transgenic plants expressing *EFR‐Cf‐9* upon treatment with elf18 variants. Quantitative Reverse Transcription Polymerase Chain Reaction (qRT PCR) was used to determine *ACRE‐132* (A) and *HIN1* (B) expression levels in WT and *EFR‐Cf‐9* expressing transgenic K1A plants upon treatment with Milli‐Q (MQ) water or with elf18C, elf18B, elf18G and flg22 at a concentration of 100 nM. RNA was extracted from infiltrated leaf sectors obtained from three separate plants for each genotype. Bars represent average ±  standard deviation (SD) of three replicates. Different letters indicate statistically significant differences, according to one‐way analysis of variance (ANOVA) followed by Tukey's significance test (*P* < 0.05). This experiment was repeated twice with similar results.

**Fig. S6** Transgenic plants expressing *EFR‐Cf‐9 *show unaltered susceptibility to *Botrytis cinerea*. Leaves of soil grown 4‐week‐old wild type (WT) and transgenic plants expressing *EFR‐Cf‐9* (K1A and K5A) were inoculated with *B. cinerea* spore suspension, and lesion areas were measured at 48 hpi. Bars indicate average lesion area ± standard error (SE) (*n* > 18). No significant difference was observed between WT and transgenic lines (K1A and K5A), according to Student's *t*‐test (*P* > 0.5)

## References

[mpp12789-bib-0001] Afroz, A. , Chaudhry, Z. , Rashid, U. , Ali, G.M. , Nazir, F. , Iqbal, J. and Khan, M.R. (2011) Enhanced resistance against bacterial wilt in transgenic tomato (*Lycopersicon esculentum*) lines expressing the *Xa21* gene. Plant Cell Tissue Organ Cult. 104, 227–237.

[mpp12789-bib-0002] Albert, M. , Jehle, A.K. , Mueller, K. , Eisele, C. , Lipschis, M. and Felix, G. (2010) *Arabidopsis thaliana* pattern recognition receptors for bacterial elongation factor Tu and flagellin can be combined to form functional chimeric receptors. J. Biol. Chem. 285, 19035–19042. 20410299 10.1074/jbc.M110.124800 PMC2885181

[mpp12789-bib-0003] Albert, I. , Böhm, H. , Albert, M. , Feiler, C.E. , Imkampe, J. , Wallmeroth, N. , Brancato, C. , Raaymakers, T.M. , Oome, S. , Zhang, H. , Krol, E. , Grefen, C. , Gust, A.A. , Chai, J. , Hedrich, R. , Van den Ackerveken, G. and Nürnberger, T. (2015) An RLP23‐SOBIR1‐BAK1 complex mediates NLP‐triggered immunity. Nature Plants, 1, 15140–15148. 27251392 10.1038/nplants.2015.140

[mpp12789-bib-0004] Bendahmane, A. , Farnham, G. , Moffett, P. and Baulcombe, D.C. (2002) Constitutive gain‐of‐function mutants in a nucleotide binding site‐leucine rich repeat protein encoded at the Rx locus of potato. Plant J. 32, 195–204. 12383085 10.1046/j.1365-313x.2002.01413.x

[mpp12789-bib-0005] Bi, G. , Liebrand, T.W.H. , Bye, R.R. , Postma, J. , van der Burgh, A.M. , Robatzek, S. , Xu, X. and Joosten, M.H.A.J. (2016) SOBIR1 requires the GxxxG dimerization motif in its transmembrane domain to form constitutive complexes with receptor‐like proteins. Mol. Plant Pathol. 17, 96–107. 25891985 10.1111/mpp.12266 PMC6638328

[mpp12789-bib-0006] Boutrot, F. and Zipfel, C. (2017) Function, discovery, and exploitation of plant pattern recognition receptors for broad‐spectrum disease resistance. Annu. Rev. Phytopathol. 55, 257–286. 28617654 10.1146/annurev-phyto-080614-120106

[mpp12789-bib-0007] Brutus, A. , Sicilia, F. , Macone, A. , Cervone, F. and De Lorenzo, G. (2010) A domain swap approach reveals a role of the plant wall‐associated kinase 1 (WAK1) as a receptor of oligogalacturonides. Proc. Natl. Acad. Sci. USA, 107, 9452–9457. 20439716 10.1073/pnas.1000675107 PMC2889104

[mpp12789-bib-0008] Böhm, H. , Albert, I. , Fan, L. , Reinhard, A. and Nürnberger, T. (2014) Immune receptor complexes at the plant cell surface. Curr. Opin. Plant Biol. 20, 47–54. 24835204 10.1016/j.pbi.2014.04.007

[mpp12789-bib-0009] Chen, W. and Ow, D.W. (2017) Precise, flexible and affordable gene stacking for crop improvement. Bioengineered, 8, 451–456. 28071976 10.1080/21655979.2016.1276679 PMC5639857

[mpp12789-bib-0010] Chen, X. , Chern, M. , Canlas, P.E. , Ruan, D. , Jiang, C. and Ronald, P.C. (2010) An ATPase promotes autophosphorylation of the pattern recognition receptor XA21 and inhibits XA21‐mediated immunity. Proc. Natl. Acad. Sci. USA, 107, 8029–8034. 20385831 10.1073/pnas.0912311107 PMC2867851

[mpp12789-bib-0011] Chen, X. , Zuo, S. , Schwessinger, B. , Chern, M. , Canlas, P.E. , Ruan, D. , Zhou, X. , Wang, J. , Daudi, A. , Petzold, C.J. , Heazlewood, J.L. and Ronald, P.C. (2014) An XA21‐associated kinase (OsSERK2) regulates immunity mediated by the XA21 and XA3 immune receptors. Mol. Plant, 7, 874–892. 24482436 10.1093/mp/ssu003 PMC4064043

[mpp12789-bib-0012] Chisholm, S.T. , Coaker, G. , Day, B. and Staskawicz, B.J. (2006) Host‐microbe interactions: shaping the evolution of the plant immune response. Cell, 124, 803–814. 16497589 10.1016/j.cell.2006.02.008

[mpp12789-bib-0013] Cook, D.E. , Mesarich, C.H. and Thomma, B.P.H.J. (2015) Understanding plant immunity as a surveillance system to detect invasion. Annu. Rev. Phytopathol. 53, 541–563. 26047564 10.1146/annurev-phyto-080614-120114

[mpp12789-bib-0014] Couto, D. and Zipfel, C. (2016) Regulation of pattern recognition receptor signalling in plants. Nature Rev. Immunol. 16, 537–552. 27477127 10.1038/nri.2016.77

[mpp12789-bib-0015] Dangl, J.L. , Horvath, D.M. and Staskawicz, B.J. (2013) Pivoting the plant immune system from dissection to deployment. Science, 341, 746–751. 23950531 10.1126/science.1236011 PMC3869199

[mpp12789-bib-0016] De Lorenzo, G. , Brutus, A. , Savatin, D.V. , Sicilia, F. and Cervone, F. (2011) Engineering plant resistance by constructing chimeric receptors that recognize damage‐associated molecular patterns (DAMPs). FEBS Lett. 585, 1521–1528. 21536040 10.1016/j.febslet.2011.04.043

[mpp12789-bib-0017] Dodds, P.N. and Rathjen, J.P. (2010) Plant immunity: towards an integrated view of plant‐pathogen interactions. Nature Rev. Genet. 11, 539–548. 20585331 10.1038/nrg2812

[mpp12789-bib-0018] Domazakis, E. , Wouters, D. , Visser, R.G.F. , Kamoun, S. , Joosten, M. and Vleeshouwers, V. (2018) The ELR‐SOBIR1 Complex Functions as a Two‐Component Receptor‐Like Kinase to Mount Defense Against Phytophthora infestans. Mol. Plant‐Microbe Interact. 31, 795–802. 29451434 10.1094/MPMI-09-17-0217-R

[mpp12789-bib-0019] Durrant, W.E. , Rowland, O. , Piedras, P. , Hammond‐Kosack, K.E. and Jones, J.D.G. (2000) cDNA‐AFLP reveals a striking overlap in race‐specific resistance and wound response gene expression profiles. Plant Cell, 12, 963–977. 10852940 10.1105/tpc.12.6.963 PMC149096

[mpp12789-bib-0020] Felix, G. , Duran, J.D. , Volko, S. and Boller, T. (1999) Plants have a sensitive perception system for the most conserved domain of bacterial flagellin. Plant J. 18, 265–276. 10377992 10.1046/j.1365-313x.1999.00265.x

[mpp12789-bib-0067] Ferrari, S. , Plotnikova, J.M. , De Lorenzo, G. and Ausubel, F.M. (2003) Arabidopsis local resistance to Botrytis cinerea involves salicylic acid and camalexin and requires EDS4 and PAD2, but not SID2, EDS5 or PAD4. Plant J. 35, 193–205. 12848825 10.1046/j.1365-313x.2003.01794.x

[mpp12789-bib-0021] Ferrari, S. , Galletti, R. , Vairo, D. , Cervone, F. and De Lorenzo, G. (2006) Antisense expression of the *Arabidopsis thaliana* *AtPGIP1* gene reduces polygalacturonase‐inhibiting protein accumulation and enhances susceptibility to *Botrytis cinerea* . Mol. Plant‐Microbe Interact. 19, 931–936. 16903359 10.1094/MPMI-19-0931

[mpp12789-bib-0022] Gopalan, S. , Wei, W. and He, S.Y. (1996) *hrp *gene‐dependent induction of *hin1*: a plant gene activated rapidly by both harpins and the *avrPto *gene‐mediated signal. Plant J. 10, 591–600. 8893538 10.1046/j.1365-313x.1996.10040591.x

[mpp12789-bib-0023] Gust, A.A. and Felix, G. (2014) Receptor like proteins associate with SOBIR1‐type of adaptors to form bimolecular receptor kinases. Curr. Opin. Plant Biol. 21, 104–111. 25064074 10.1016/j.pbi.2014.07.007

[mpp12789-bib-0024] Gómez‐Gómez, L. and Boller, T. (2000) FLS2: an LRR receptor‐like kinase involved in the perception of the bacterial elicitor flagellin in *Arabidopsis* . Mol. Cell 5, 1003–1011. 10911994 10.1016/s1097-2765(00)80265-8

[mpp12789-bib-0025] Halpin, C. (2005) Gene stacking in transgenic plants‐the challenge for 21st century plant biotechnology. Plant Biotechnol. J. 3, 141–155. 17173615 10.1111/j.1467-7652.2004.00113.x

[mpp12789-bib-0026] Hammond‐Kosack, K.E. , Tang, S. , Harrison, K. and Jones, J.D.G. (1998) The tomato *Cf‐9* disease resistance gene functions in tobacco and potato to confer responsiveness to the fungal avirulence gene product Avr 9. Plant Cell, 10, 1251–1266. 9707527 10.1105/tpc.10.8.1251 PMC144066

[mpp12789-bib-0027] He, Z. , Wang, Z. , Li, J. , Zhu, Q. , Lamb, C. , Ronald, P. and Chory, J. (2000) Perception of brassinosteroids by the extracellular domain of the receptor kinase BRI1. Science, 288, 2360–2363 10875920 10.1126/science.288.5475.2360

[mpp12789-bib-0028] Heese, A. , Hann, D.R. , Gimenez‐Ibanez, S. , Jones, A.M. , He, K. , Li, J. , Schroeder, J.I. , Peck, S.C. and Rathjen, J.P. (2007) The receptor‐like kinase SERK3/BAK1 is a central regulator of innate immunity in plants. Proc. Natl. Acad. Sci. USA, 104, 12217–12222. 17626179 10.1073/pnas.0705306104 PMC1924592

[mpp12789-bib-0029] Hegenauer, V. , Furst, U. , Kaiser, B. , Smoker, M. , Zipfel, C. , Felix, G. , Stahl, M. and Albert, M. (2016) Detection of the plant parasite *Cuscuta reflexa* by a tomato cell surface receptor. Science, 353, 478–481. 27471302 10.1126/science.aaf3919

[mpp12789-bib-0030] Higuchi, R. , Krummel, B. and Saiki, R.K. (1988) A general method of in vitro preparation and specific mutagenesis of DNA fragments: study of protein and DNA interactions. Nucleic Acids Res. 16, 7351–7367. 3045756 10.1093/nar/16.15.7351 PMC338413

[mpp12789-bib-0031] Holton, N. , Nekrasov, V. , Ronald, P.C. and Zipfel, C. (2015) The phylogenetically‐related pattern recognition receptors EFR and XA21 recruit similar immune signaling components in monocots and dicots. PLoS Pathog. 11, e1004602. 25607985 10.1371/journal.ppat.1004602 PMC4301810

[mpp12789-bib-0032] Horsch, R.B. , Fraley, R.T. , Rogers, S.G. , Klee, H.J. , Fry, J. , Hinchee, M.A. and Shah, D.S. (1988) *Agrobacterium*‐mediated gene transfer to plants; engineering tolerance to glyphosate. Iowa State J. Res. 62, 487–502.

[mpp12789-bib-0033] Jones, D.A. , Thomas, C.M. , Hammond‐Kosack, K.E. , Balint‐Kurti, P.J. and Jones, J.D.G. (1994) Isolation of the tomato *Cf‐9* gene for resistance to *Cladosporium fulvum* by transposon tagging. Science, 266, 789–793. 7973631 10.1126/science.7973631

[mpp12789-bib-0034] Jones, J.D.G. and Dangl, J.L. (2006) The plant immune system. Nature, 444, 323–329. 17108957 10.1038/nature05286

[mpp12789-bib-0035] Joosten, M.H.A.J. , Vogelsang, R. , Cozijnsen, T.J. , Verberne, M.C. and de Wit, P.J.G.M. (1997) The biotrophic fungus *Cladosporium fulvum* circumvents *Cf‐4*‐mediated resistance by producing unstable AVR4 elicitors. Plant Cell, 9, 367–379. 9090881 10.1105/tpc.9.3.367 PMC156924

[mpp12789-bib-0036] van Kan, J.A.L. , van den Ackerveken, G.F.J.M. and de Wit, P.J.G.M. (1991) Cloning and characterization of cDNA of avirulence gene *avr9 *of the fungal pathogen *Cladosporium fulvum*, causal agent of tomato leaf mold. Mol. Plant‐Microbe Interact. 4, 52–59. 1799694 10.1094/mpmi-4-052

[mpp12789-bib-0037] Kishimoto, K. , Kouzai, Y. , Kaku, H. , Shibuya, N. , Minami, E. and Nishizawa, Y. (2010) Perception of the chitin oligosaccharides contributes to disease resistance to blast fungus *Magnaporthe oryzae* in rice. Plant J. 64, 343–354. 21070413 10.1111/j.1365-313X.2010.04328.x

[mpp12789-bib-0038] Kouzai, Y. , Kaku, H. , Shibuya, N. , Minami, E. and Nishizawa, Y. (2013) Expression of the chimeric receptor between the chitin elicitor receptor CEBiP and the receptor‐like protein kinase Pi‐d2 leads to enhanced responses to the chitin elicitor and disease resistance against *Magnaporthe oryzae* in rice. Plant Mol. Biol. 81, 287–295. 23242918 10.1007/s11103-012-9998-7

[mpp12789-bib-0039] Kunze, G. , Zipfel, C. , Robatzek, S. , Niehaus, K. , Boller, T. and Felix, G. (2004) The N terminus of bacterial elongation factor Tu elicits innate immunity in Arabidopsis plants. Plant Cell, 16, 3496–3507. 15548740 10.1105/tpc.104.026765 PMC535888

[mpp12789-bib-0040] Lacombe, S. , Rougon‐Cardoso, A. , Sherwood, E. , Peeters, N. , Dahlbeck, D. , van Esse, H.P. , Smoker, M. , Rallapalli, G. , Thomma, B.P.H.J. , Staskawicz, B. , Jones, J.D.G. and Zipfel, C. (2010) Interfamily transfer of a plant pattern‐recognition receptor confers broad‐spectrum bacterial resistance. Nature Biotechnol. 28, 365–369. 20231819 10.1038/nbt.1613

[mpp12789-bib-0041] Lee, S. , Yang, D.S. , Uppalapati, S.R. , Sumner, L.W. and Mysore, K.S. (2013) Suppression of plant defense responses by extracellular metabolites from *Pseudomonas syringae* pv. *tabaci *in *Nicotiana benthamiana* . BMC Plant Biol. 13, 65. 23597256 10.1186/1471-2229-13-65 PMC3648423

[mpp12789-bib-0042] Liebrand, T.W.H. , Smit, P. , Abd‐El‐Haliem, A.M. , de Jonge, R. , Cordewener, J.H.G. , America, A.H.P. , Sklenar, J. , Jones, A.M. , Robatzek, S. , Thomma, B.P. and Tameling, W.I. (2012) Endoplasmic reticulum‐quality control chaperones facilitate the biogenesis of Cf receptor‐like proteins involved in pathogen resistance of tomato. Plant Physiol. 159, 1819–1833. 22649272 10.1104/pp.112.196741 PMC3425215

[mpp12789-bib-0043] Liebrand, T.W.H. , van den Berg, G.C.M. , Zhang, Z. , Smit, P. , Cordewener, J.H.G. , America, A.H.P. , Sklenar, J. , Jones, A.M. , Tameling, W.I. , Robatzek, S. and Thomma, B.P. (2013) Receptor‐like kinase SOBIR1/EVR interacts with receptor‐like proteins in plant immunity against fungal infection. Proc. Natl. Acad. Sci. USA, 110, 10010–10015. 23716655 10.1073/pnas.1220015110 PMC3683720

[mpp12789-bib-0044] Liebrand, T.W.H. , van den Burg, H.A. and Joosten, M.H.A.J. (2014) Two for all: receptor‐associated kinases SOBIR1 and BAK1. Trends Plant Sci. 19, 123–132. 24238702 10.1016/j.tplants.2013.10.003

[mpp12789-bib-0045] Ma, L. and Borhan, M.H. (2015) The receptor‐like kinase SOBIR1 interacts with *Brassica napus* LepR3 and is required for *Leptosphaeria maculans* AvrLm1‐triggered immunity. Front. Plant Sci. 6, 933 10.3389/fpls.2015.00933 26579176 PMC4625043

[mpp12789-bib-0046] Manfredini, C. , Sicilia, F. , Ferrari, S. , Pontiggia, D. , Salvi, G. , Caprari, C. , Lorito, M. and De Lorenzo, G. (2005) Polygalacturonase‐inhibiting protein 2 of *Phaseolus vulgaris* inhibits BcPG1, a polygalacturonase of *Botrytis cinerea* important for pathogenicity, and protects transgenic plants from infection. Physiol. Mol. Plant Pathol. 67, 108–115.

[mpp12789-bib-0047] Mattei, B. , Spinelli, F. , Pontiggia, D. and De Lorenzo, G. (2016) Comprehensive Analysis of the Membrane Phosphoproteome Regulated by Oligogalacturonides in Arabidopsis thaliana. Front. Plant Sci. 7, 1107 10.3389/fpls.2016.01107 27532006 PMC4969306

[mpp12789-bib-0048] Mendes, B. , Cardoso, S. , Boscariol‐Camargo, R. , Cruz, R. , Mourão Filho, F. and Bergamin Filho, A. (2010) Reduction in susceptibility to *Xanthomonas axonopodis* pv. *citri *in transgenic *Citrus sinensis* expressing the rice *Xa21 *gene. Plant Pathol. 59, 68–75.

[mpp12789-bib-0049] Postma, J. , Liebrand, T.W.H. , Bi, G. , Evrard, A. , Bye, R.R. , Mbengue, M. , Kuhn, H. , Joosten, M.H.A.J. and Robatzek, S. (2016) Avr4 promotes Cf‐4 receptor‐like protein association with the BAK1/SERK3 receptor‐like kinase to initiate receptor endocytosis and plant immunity. New Phytol. 210, 627–642. 26765243 10.1111/nph.13802

[mpp12789-bib-0050] Roux, M. , Schwessinger, B. , Albrecht, C. , Chinchilla, D. , Jones, A. , Holton, N. , Malinovsky, F.G. , Tör, M. , de Vries, S. and Zipfel, C. (2011) The *Arabidopsis *leucine‐rich repeat receptor‐like kinases BAK1/SERK3 and BKK1/SERK4 are required for innate immunity to hemibiotrophic and biotrophic pathogens. Plant Cell, 23, 2440–2455. 21693696 10.1105/tpc.111.084301 PMC3160018

[mpp12789-bib-0051] Ruijter, J.M. , Ramakers, C. , Hoogaars, W.M.H. , Karlen, Y. , Bakker, O. , van den Hoff, M.J.B. and Moorman, A.F.M. (2009) Amplification efficiency: linking baseline and bias in the analysis of quantitative PCR data. Nucleic Acids Res. 37, e45. 19237396 10.1093/nar/gkp045 PMC2665230

[mpp12789-bib-0052] Schoonbeek, H.J. , Wang, H.H. , Stefanato, F.L. , Craze, M. , Bowden, S. , Wallington, E. , Zipfel, C. and Ridout, C.J. (2015) Arabidopsis EF‐Tu receptor enhances bacterial disease resistance in transgenic wheat. New Phytol. 206, 606–613. 25760815 10.1111/nph.13356

[mpp12789-bib-0053] Schwessinger, B. , Bahar, O. , Thomas, N. , Holton, N. , Nekrasov, V. , Ruan, D. , Canlas, P.E. , Daudi, A. , Petzold, C.J. , Singan, V.R. and Kuo, R. (2015) Transgenic expression of the dicotyledonous pattern recognition receptor EFR in rice leads to ligand‐dependent activation of defense responses. PLoS Pathog. 11, e1004809. 25821973 10.1371/journal.ppat.1004809 PMC4379099

[mpp12789-bib-0054] Schwessinger, B. , Roux, M. , Kadota, Y. , Ntoukakis, V. , Sklenar, J. , Jones, A. and Zipfel, C. (2011) Phosphorylation‐dependent differential regulation of plant growth, cell death, and innate immunity by the regulatory receptor‐like kinase BAK1. PLoS Genet. 7, e1002046. 21593986 10.1371/journal.pgen.1002046 PMC3085482

[mpp12789-bib-0055] Song, Y. , Liu, L. , Wang, Y. , Valkenburg, D.J. , Zhang, X. , Zhu, L. and Thomma, B.P. (2017) Transfer of tomato immune receptor Ve1 confers Ave1‐dependent *Verticillium *resistance in tobacco and cotton. Plant Biotechnol. J. 16, 638–648. 28796297 10.1111/pbi.12804 PMC5787823

[mpp12789-bib-0056] Stergiopoulos, I. and de Wit, P.J.G.M. (2009) Fungal effector proteins. Annu. Rev. Phytopathol. 47, 233–263. 19400631 10.1146/annurev.phyto.112408.132637

[mpp12789-bib-0057] Thomas, C.M. , Jones, D.A. , Parniske, M. , Harrison, K. , Balint‐Kurti, P.J. , Hatzixanthis, K. and Jones, J.D. (1997) Characterization of the tomato *Cf‐4* gene for resistance to *Cladosporium fulvum* identifies sequences that determine recognitional specificity in Cf‐4 and Cf‐9. Plant Cell, 9, 2209–2224. 9437864 10.1105/tpc.9.12.2209 PMC157069

[mpp12789-bib-0058] Thomas, N.C. , Oksenberg, N. , Liu, F. , Caddell, D. , Nalyvayko, A. , Nguyen, Y. , Schwessinger, B. and Ronald, P.C. (2017) The rice XA21 ectodomain fused to the Arabidopsis EFR cytoplasmic domain confers resistance to *Xanthomonas oryzae* pv. *oryzae* . Peer J. 6, e4456. 10.7717/peerj.4456 PMC5949059 29761034

[mpp12789-bib-0059] Tripathi, J.N. , Lorenzen, J. , Bahar, O. , Ronald, P. and Tripathi, L. (2014) Transgenic expression of the rice *Xa21* pattern‐recognition receptor in banana (*Musa *sp.) confers resistance to *Xanthomonas campestris* pv. *musacearum* . Plant Biotechnol. J. 12, 663–673. 24612254 10.1111/pbi.12170 PMC4110157

[mpp12789-bib-0060] Van Der Burgh, A.M. , Postma, J. , Robatzek, S. and Joosten, M. (2018) Kinase activity of SOBIR1 and BAK1 is required for immune signalling. Mol. Plant Pathol. 20, 410–422. 10.1111/mpp.12767 PMC6637861 30407725

[mpp12789-bib-0061] Van der Hoorn, R.A. , Laurent, F. , Roth, R. and De Wit, P.J. (2000) Agroinfiltration is a versatile tool that facilitates comparative analyses of *Avr9*/*Cf‐9*‐induced and *Avr4*/*Cf‐4*‐induced necrosis. Mol. Plant‐Microbe Interact. 13, 439–446. 10755307 10.1094/MPMI.2000.13.4.439

[mpp12789-bib-0500] Wang, Y. , Xu, Y. , Sun, Y. , Wang, H. , Qi, J. , Wan, B. , Ye, W. , Lin, Y. , Shao, Y. , Dong, S. , Tyler, B.M. and Wang, Y. (2018) Leucine‐rich repeat receptor‐like gene screen reveals that *Nicotiana* RXEG1 regulates glycoside hydrolase 12 MAMP detection. Nat. Commun. 9, 594 10.1038/s41467-018-03010-8 29426870 PMC5807360

[mpp12789-bib-0062] Zhang, W. , Fraiture, M. , Kolb, D. , Loffelhardt, B. , Desaki, Y. , Boutrot, F.F.G. , Tor, M. , Zipfel, C. , Gust, A.A. and Brunner, F. (2013) *Arabidopsis *receptor‐like protein30 and receptor‐like kinase suppressor of BIR1‐1/EVERSHED mediate innate immunity to necrotrophic fungi. Plant Cell, 25, 4227–4241. 24104566 10.1105/tpc.113.117010 PMC3877809

[mpp12789-bib-0063] Zhang, L. , Kars, I. , Essenstam, B. , Liebrand, T.W.H. , Wagemakers, L. , Elberse, J. , Tagkalaki, P. , Tjoitang, D. , van den Ackerveken, G. and van Kan, J.A.L. (2014) Fungal endopolygalacturonases are recognized as microbe‐associated molecular patterns by the *Arabidopsis *receptor‐like protein RESPONSIVENESS TO BOTRYTIS POLYGALACTURONASES1. Plant Physiol. 164, 352–364. 24259685 10.1104/pp.113.230698 PMC3875813

[mpp12789-bib-0064] Zhu, S. , Li, Y. , Vossen, J.H. , Visser, R.G.F. and Jacobsen, E. (2012) Functional stacking of three resistance genes against *Phytophthora infestans* in potato. Transgenic Res. 21, 89–99. 21479829 10.1007/s11248-011-9510-1 PMC3264857

[mpp12789-bib-0065] Zipfel, C. (2014) Plant pattern‐recognition receptors. Trends Immunol. 35, 345–351. 24946686 10.1016/j.it.2014.05.004

[mpp12789-bib-0066] Zipfel, C. , Kunze, G. , Chinchilla, D. , Caniard, A. , Jones, J.D.G. , Boller, T. and Felix, G. (2006) Perception of the bacterial PAMP EF‐Tu by the receptor EFR restricts *Agrobacterium*‐mediated transformation. Cell, 125, 749–760. 16713565 10.1016/j.cell.2006.03.037

