# Supplementary material for: An EFR‐Cf‐9 chimera confers enhanced resistance to bacterial pathogens by SOBIR1‐ and BAK1‐dependent recognition of elf18
Source: Mol Plant Pathol. 2019 Apr 1;20(6):751–64. doi: 10.1111/mpp.12789 (PMC6637901; doi:10.1111/mpp.12789)
Supplement: Supplementary file 1 — Fig. S1 EFR‐Cf‐9 does not trigger an HR in tobacco plants expressing Avr9. eGFP‐tagged EFR, Cf‐9 and EFR‐Cf‐9 were transiently expressed in Avr9‐transgenic N. tabacum SR1 plants at a final OD600 of 1.0. For each construct, at least three leaves, taken from separate plants, were agro‐infiltrated. Pictures were taken at three days post‐infiltration (dpi). The infiltrated areas are indicated by white dashed lines. Under these conditions, all leaves agroinfiltrated with Cf‐9‐eGFP, and none of the other samples, showed necrosis of at least half of the infiltrated area. This experiment was repeated three times with similar results. Representative images are shown. [file MPP-20-751-s001.docx]

water

EFR-eGFP

Cf-9-eGFP

EFR-Cf-9-eGFP

*Avr9* transgenic *N. tabacum*


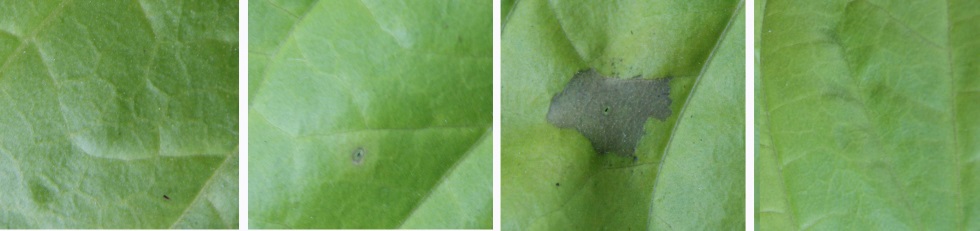


**Fig. S1. EFR-Cf-9 does not trigger an HR in tobacco plants expressing *Avr9*.** eGFP-tagged EFR, Cf-9 and EFR-Cf-9 were transiently expressed in *Avr9*-transgenic *N. tabacum* SR1 plants at a final OD_600_ of 1.0. For each construct, at least three leaves, taken from separate plants, were agro-infiltrated. Pictures were taken at three days post-infiltration (dpi). The infiltrated areas are indicated by white dashed lines. Under these conditions, all leaves agroinfiltrated with Cf-9-eGFP, and none of the other samples, showed necrosis of at least half of the infiltrated area. This experiment was repeated three times with similar results. Representative images are shown.
