# Supplementary material for: An EFR‐Cf‐9 chimera confers enhanced resistance to bacterial pathogens by SOBIR1‐ and BAK1‐dependent recognition of elf18
Source: Mol Plant Pathol. 2019 Apr 1;20(6):751–64. doi: 10.1111/mpp.12789 (PMC6637901; doi:10.1111/mpp.12789)
Supplement: Supplementary file 2 — Fig. S2 FLS2‐Cf‐9 does not trigger an HR in tobacco plants upon treatment with flg22. eGFP‐tagged FLS2, Cf‐9 and FLS2‐Cf‐9 were either expressed alone (first and third column) or transiently co‐expressed with Avr9 (second column), or, followed by treatment with Milli‐Q (MQ) water (first column) or 100 μM flg22 (third column) after two days. Agrobacteria driving expression of the various constructs were infiltrated at a final OD600 of 1.0. Pictures were taken at four days post‐infiltration (dpi) with the elicitor or at seven days of co‐expression with Avr9. The infiltrated areas are indicated by white dashed lines. For each treatment, at least three leaves per construct, taken from separate plants, were infiltrated. Under these conditions, all leaves co‐expressing Avr9 and Cf‐9‐eGFP showed necrosis of at least half of the infiltrated area, whereas none of the other samples showed HR‐like symptoms. This experiment was repeated three times with similar results. Representative images are shown. [file MPP-20-751-s002.docx]

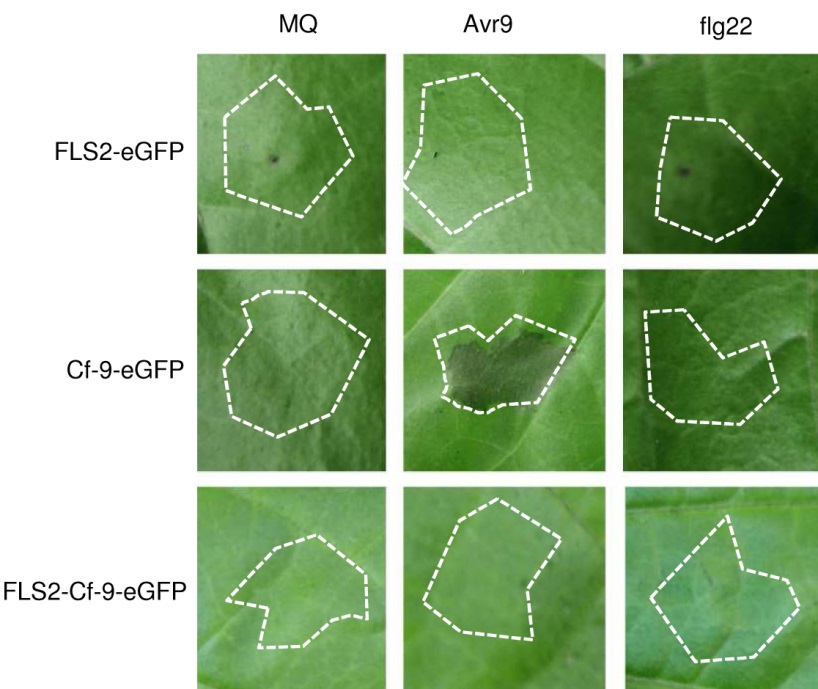


**Fig. S2. FLS2-Cf-9 does not trigger an HR in tobacco plants upon treatment with flg22.** eGFP-tagged FLS2, Cf-9 and FLS2-Cf-9 were either expressed alone (first and third column) or transiently co-expressed with Avr9 (second column), or, followed by treatment with Milli-Q (MQ) water (first column) or 100 μM flg22 (third column) after two days. Agrobacteria driving expression of the various constructs were infiltrated at a final OD_600_ of 1.0. Pictures were taken at four days post infiltration (dpi) with the elicitor or at seven days of co-expression with Avr9. The infiltrated areas are indicated by white dashed lines. For each treatment, at least three leaves per construct, taken from separate plants, were infiltrated. Under these conditions, all leaves co-expressing Avr9 and Cf-9-eGFP showed necrosis of at least half of the infiltrated area, whereas none of the other samples showed HR-like symptoms. This experiment was repeated three times with similar results. Representative images are shown.
