# Supplementary material for: An EFR‐Cf‐9 chimera confers enhanced resistance to bacterial pathogens by SOBIR1‐ and BAK1‐dependent recognition of elf18
Source: Mol Plant Pathol. 2019 Apr 1;20(6):751–64. doi: 10.1111/mpp.12789 (PMC6637901; doi:10.1111/mpp.12789)
Supplement: Supplementary file 3 — Fig. S3 Identification of EFR Cf 9 in microsomal fractions of transgenic tobacco plants. Amino acid sequence of the EFR Cf 9 chimeric receptor. The five peptides identified by mass spectrometry in microsomal protein fractions extracted from transgenic EFR‐Cf‐9 expressing K1A leaves are underlined. SP, signal peptide; LRR, leucine rich repeat; eJM, external juxtamembrane; TM, transmembrane; iJM, internal juxtamembrane. [file MPP-20-751-s003.docx]

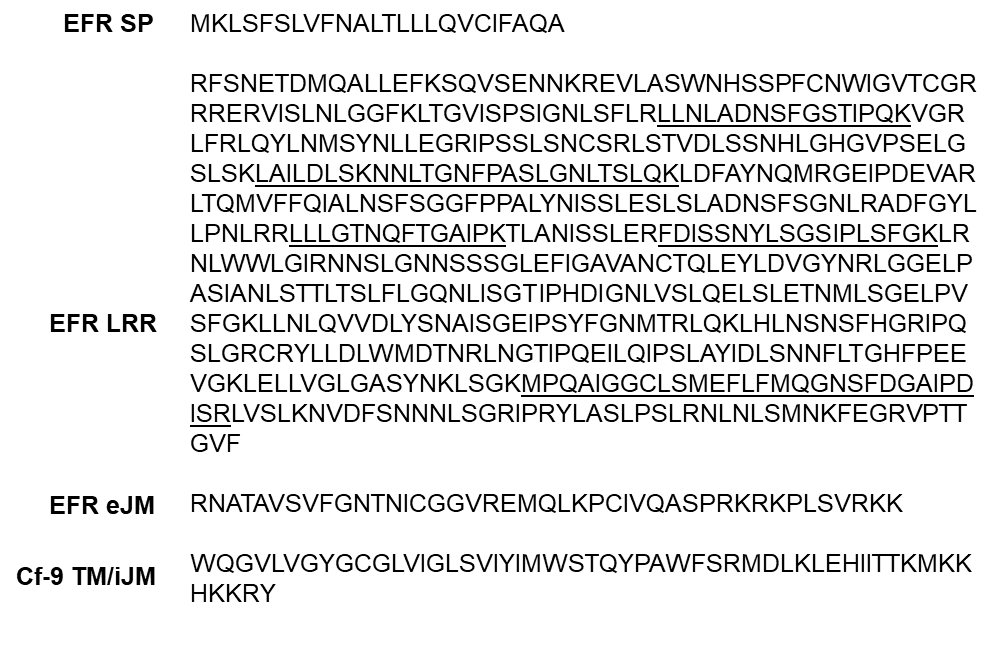


**Fig. S3. Identification of EFR-Cf-9 in microsomal fractions of transgenic tobacco plants.** Amino acid sequence of the EFR-Cf-9 chimeric receptor. The five peptides identified by mass spectrometry in microsomal protein fractions extracted from transgenic *EFR-Cf-9*-expressing K1A leaves are underlined. SP, signal peptide; LRR, leucine-rich repeat; eJM, external juxtamembrane; TM, transmembrane; iJM, internal juxtamembrane.
