# Supplementary material for: An EFR‐Cf‐9 chimera confers enhanced resistance to bacterial pathogens by SOBIR1‐ and BAK1‐dependent recognition of elf18
Source: Mol Plant Pathol. 2019 Apr 1;20(6):751–64. doi: 10.1111/mpp.12789 (PMC6637901; doi:10.1111/mpp.12789)
Supplement: Supplementary file 4 — Fig. S4 EFR Cf 9 transgenic plants recognize different elf18 variants. Fully expanded leaves of 4‐week‐old untransformed wild type (WT) and transgenic tobacco plants expressing EFR Cf 9 (K1A) plants were treated with Milli‐Q (MQ) water or with flg22, elf18C, elf18B or elf18G at a concentration of 100 nM. For each peptide, at least four leaves per genotype, taken from independent plants, were infiltrated. Pictures were taken at 40 h post‐infiltration. The infiltrated area is indicated by the white dashed line. At this time point, all leaves from K1A plants infiltrated with el18C, elf18B or elf18C showed necrosis of at least half of the infiltrated area, whereas those infiltrated with flg22 or water, and all leaves from WT plants, failed to display any HR‐like symptoms. This experiment was repeated three times with similar results, and representative images are shown. [file MPP-20-751-s004.docx]

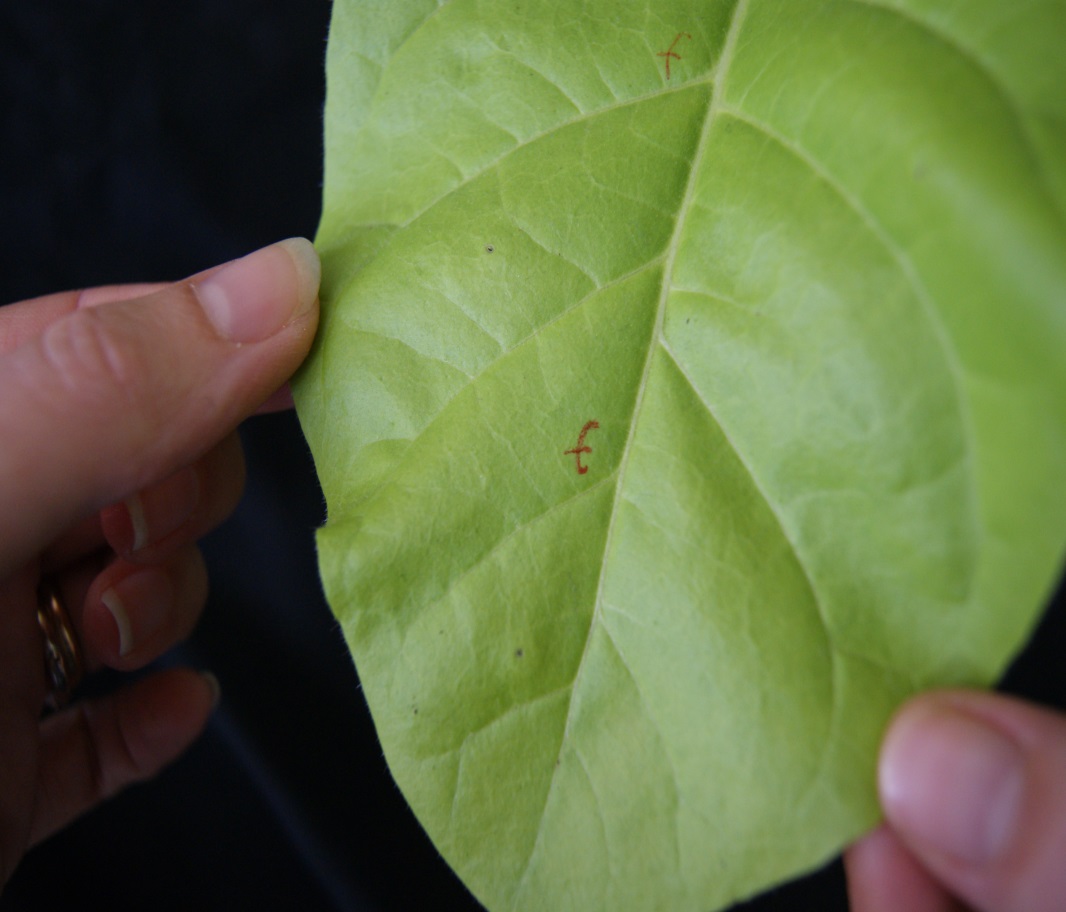

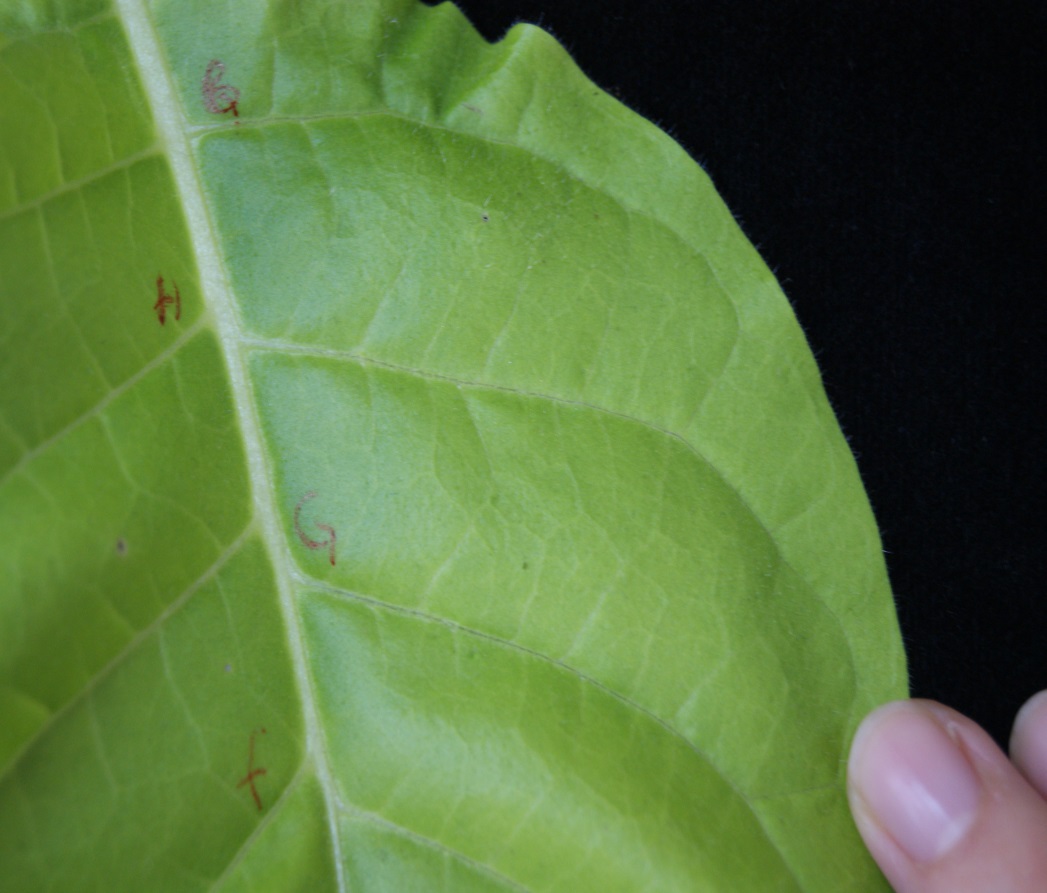

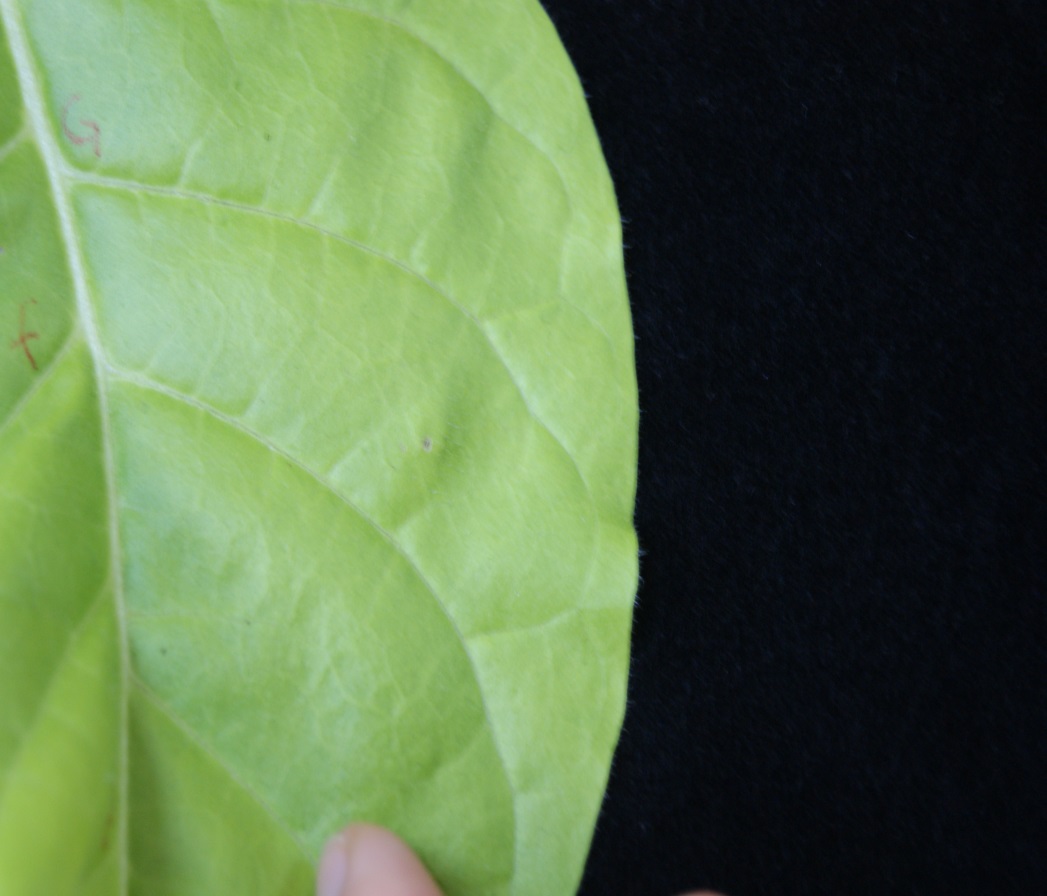

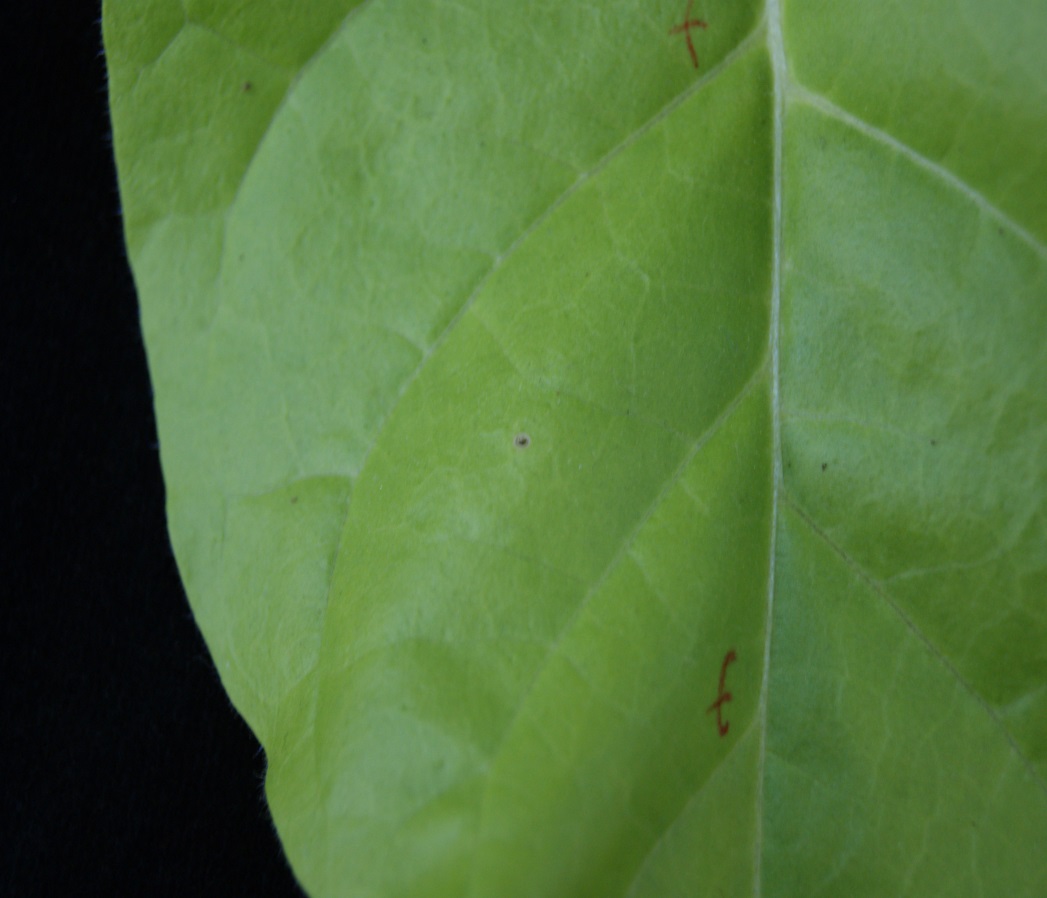

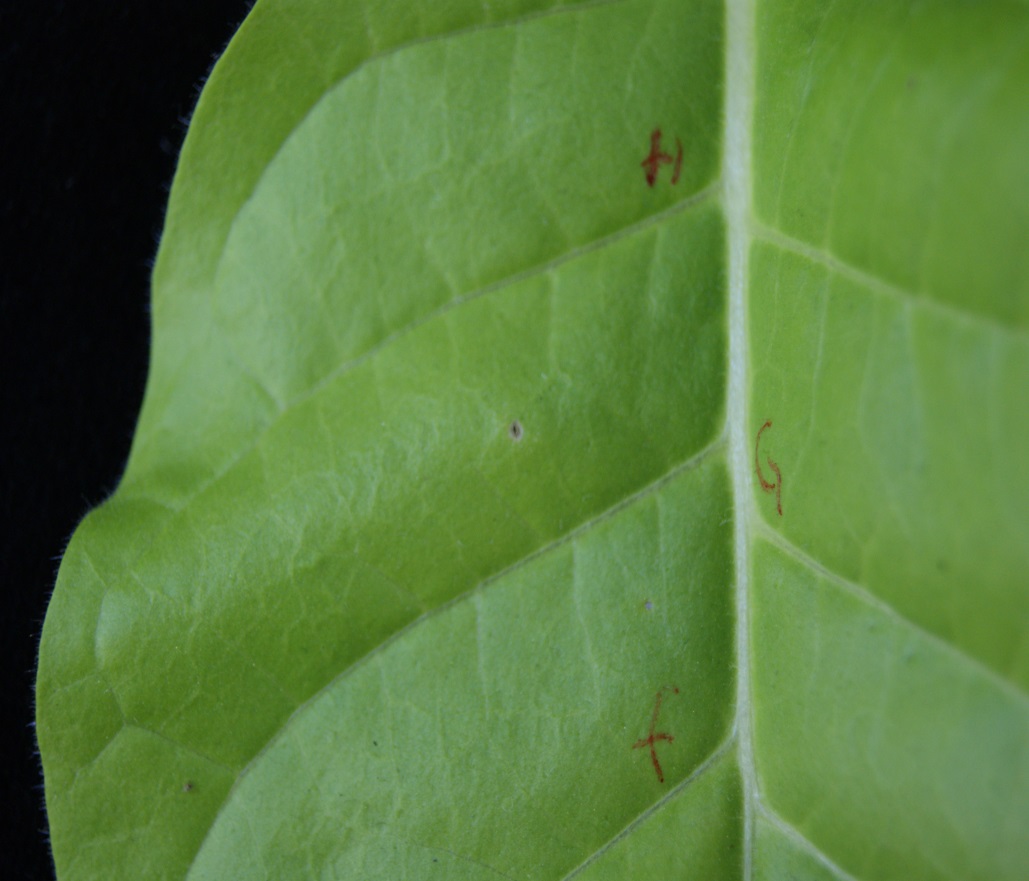

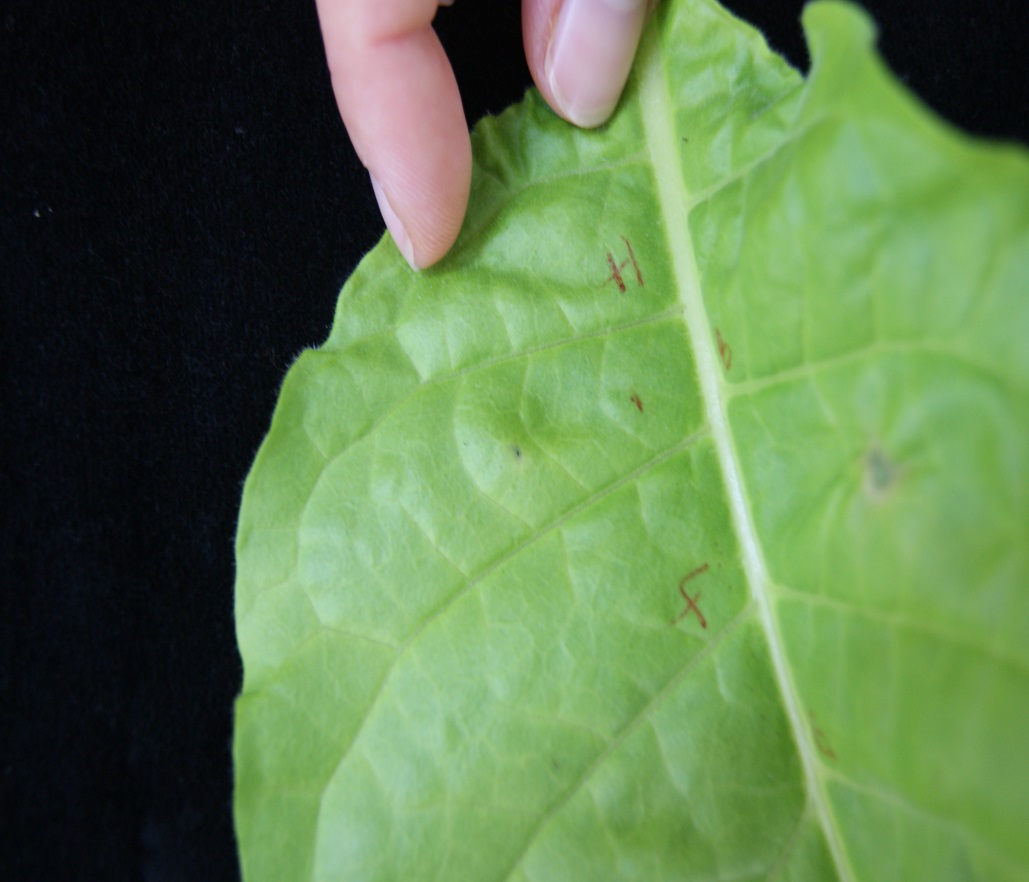

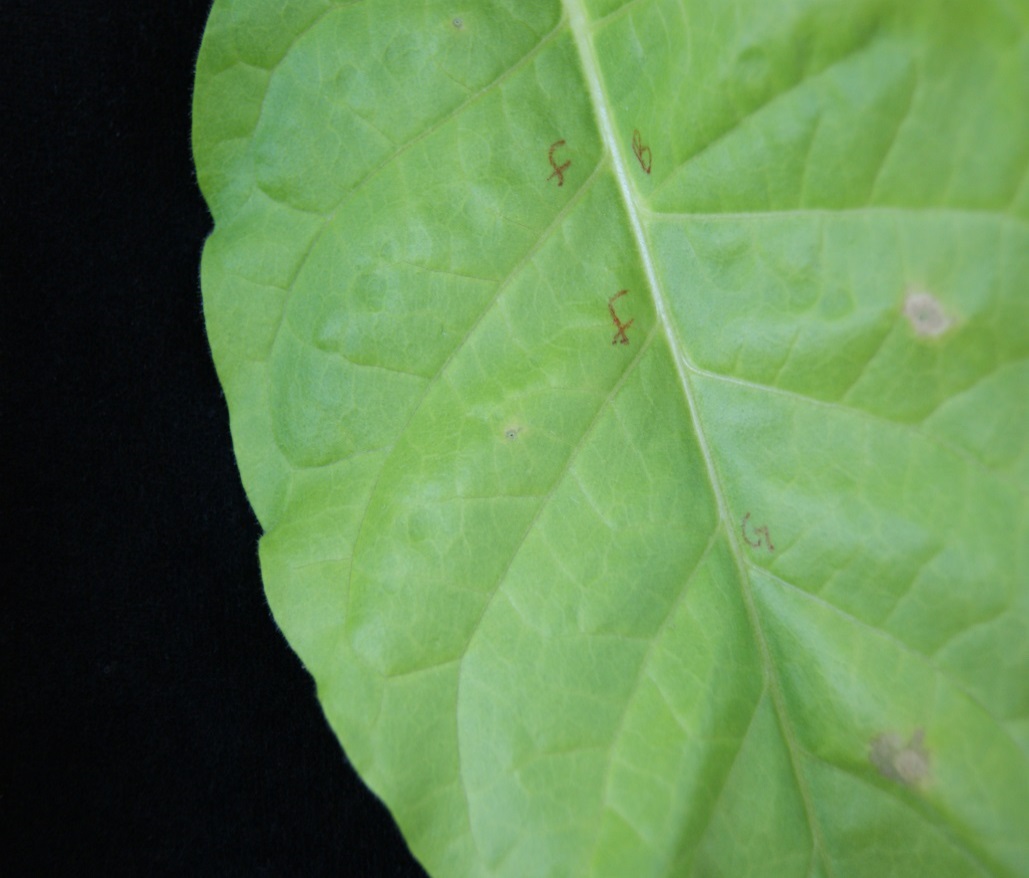

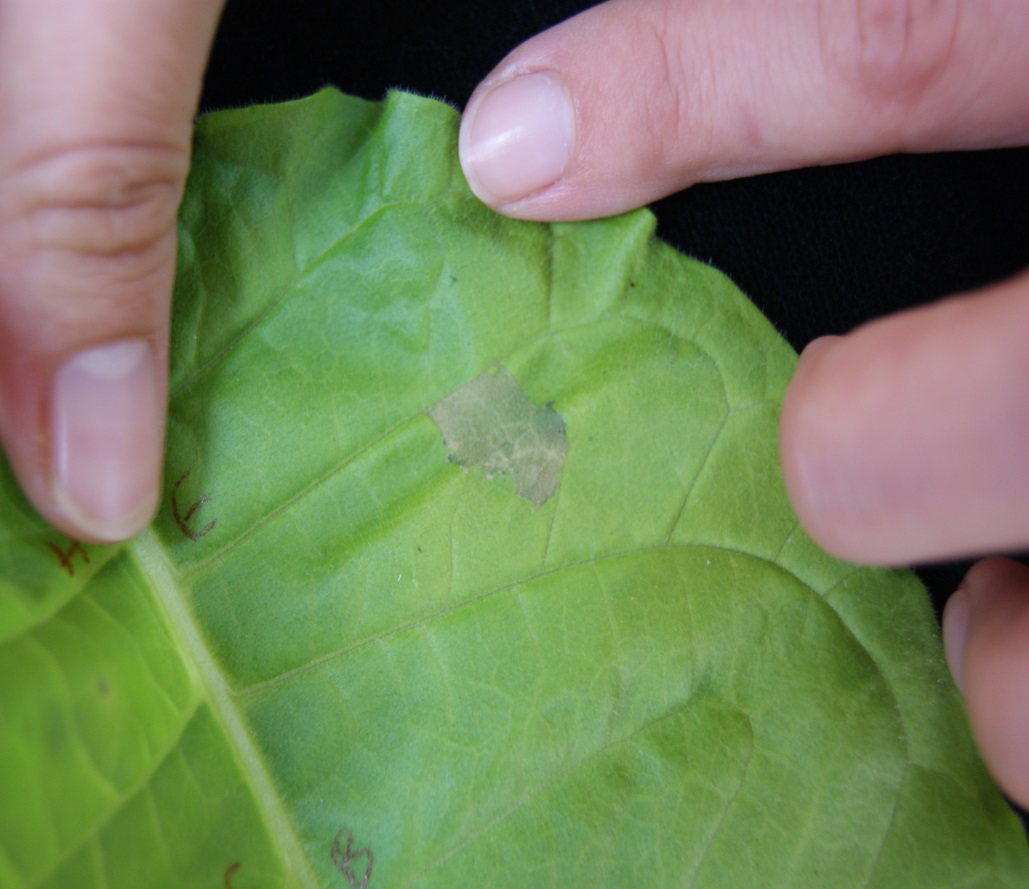

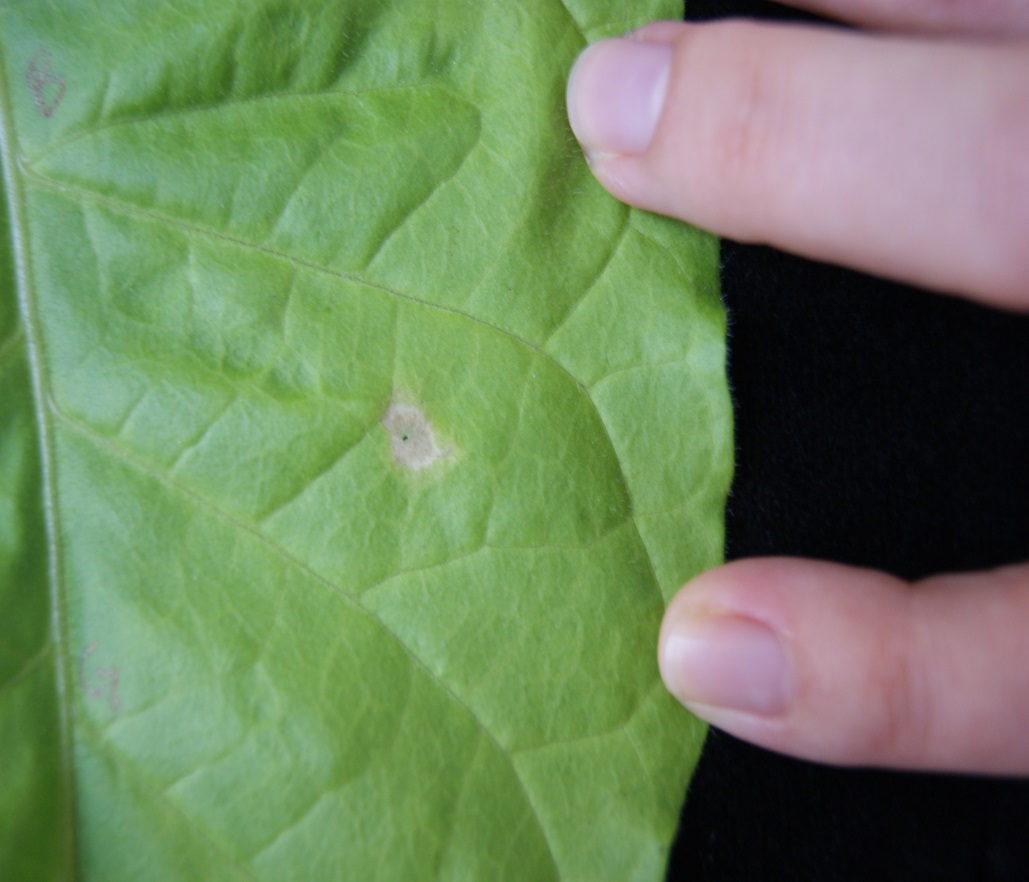

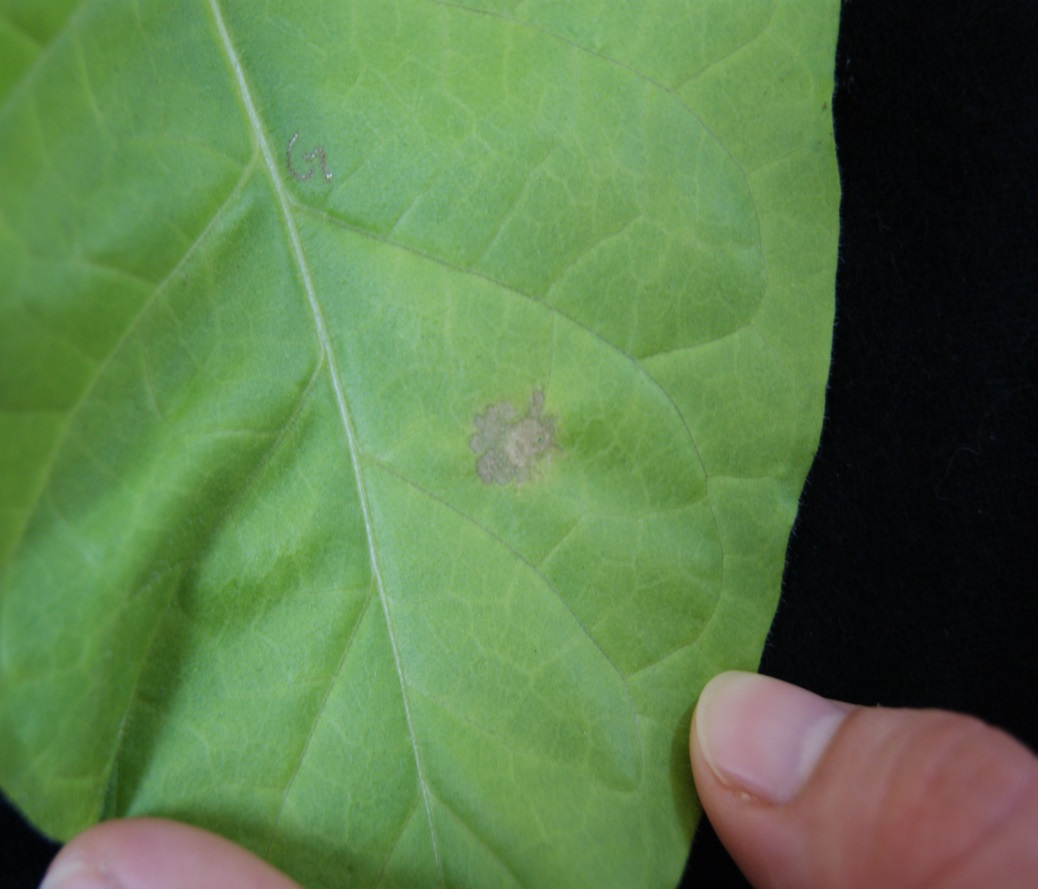


MQ

flg22

elf18C

elf18B

elf18G

K1A

WT

**Fig. S4. EFR-Cf-9-transgenic plants recognize different elf18 variants.** Fully expanded leaves of four-week-old untransformed (WT) and transgenic tobacco plants expressing EFR-Cf-9 (K1A) plants were treated with Milli-Q (MQ) water or with flg22, elf18C, elf18B or elf18G at a concentration of 100 nM. For each peptide, at least four leaves per genotype, taken from independent plants, were infiltrated. Pictures were taken at 40 hours post infiltration. The infiltrated area is indicated by the white dashed line. At this time point, all leaves from K1A plants infiltrated with el18C, elf18B or elf18C showed necrosis of at least half of the infiltrated area, whereas those infiltrated with flg22 or water, and all leaves from WT plants, failed to display any HR-like symptoms. This experiment was repeated three times with similar results, and representative images are shown.
