# Supplementary material for: An EFR‐Cf‐9 chimera confers enhanced resistance to bacterial pathogens by SOBIR1‐ and BAK1‐dependent recognition of elf18
Source: Mol Plant Pathol. 2019 Apr 1;20(6):751–64. doi: 10.1111/mpp.12789 (PMC6637901; doi:10.1111/mpp.12789)
Supplement: Supplementary file 5 — Fig. S5 Defence‐related genes ACRE‐132 and HIN1 are differentially up regulated in transgenic plants expressing EFR‐Cf‐9 upon treatment with elf18 variants. Quantitative Reverse Transcription Polymerase Chain Reaction (qRT PCR) was used to determine ACRE‐132 (A) and HIN1 (B) expression levels in WT and EFR‐Cf‐9 expressing transgenic K1A plants upon treatment with Milli‐Q (MQ) water or with elf18C, elf18B, elf18G and flg22 at a concentration of 100 nM. RNA was extracted from infiltrated leaf sectors obtained from three separate plants for each genotype. Bars represent average ± standard deviation (SD) of three replicates. Different letters indicate statistically significant differences, according to one‐way analysis of variance (ANOVA) followed by Tukey's significance test (P < 0.05). This experiment was repeated twice with similar results. [file MPP-20-751-s005.docx]

**Fig. S5. Defense-related genes *ACRE132* and *HIN1* are differentially up-regulated in transgenic plants expressing *EFR-Cf-9* upon treatment with elf18 variants.** Quantitative RT-PCR was used to determine *ACRE-132* (A) and *HIN1* (B) expression levels in WT and *EFR-Cf-9*-expressing transgenic K1A plants upon treatment with Milli-Q (MQ) water or with elf18C, elf18B, elf18G and flg22 at a concentration of 100 nM. RNA was extracted from infiltrated leaf sectors obtained from three separate plants for each genotype. Bars represent average ± SD of three replicates. Different letters indicate statistically significant differences, according to one-way ANOVA followed by Tukey’s significance test (p<0.05). This experiment was repeated twice with similar results.


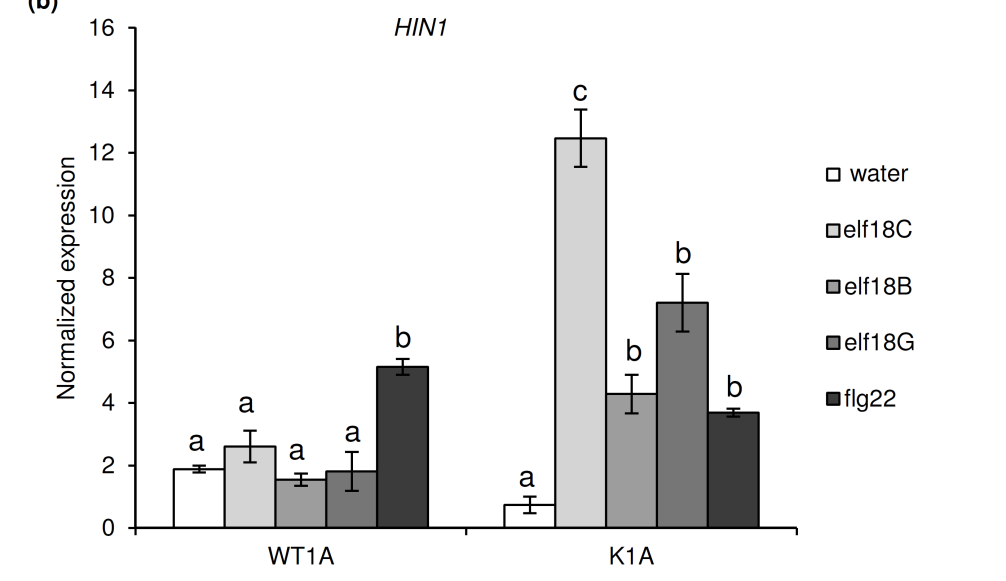

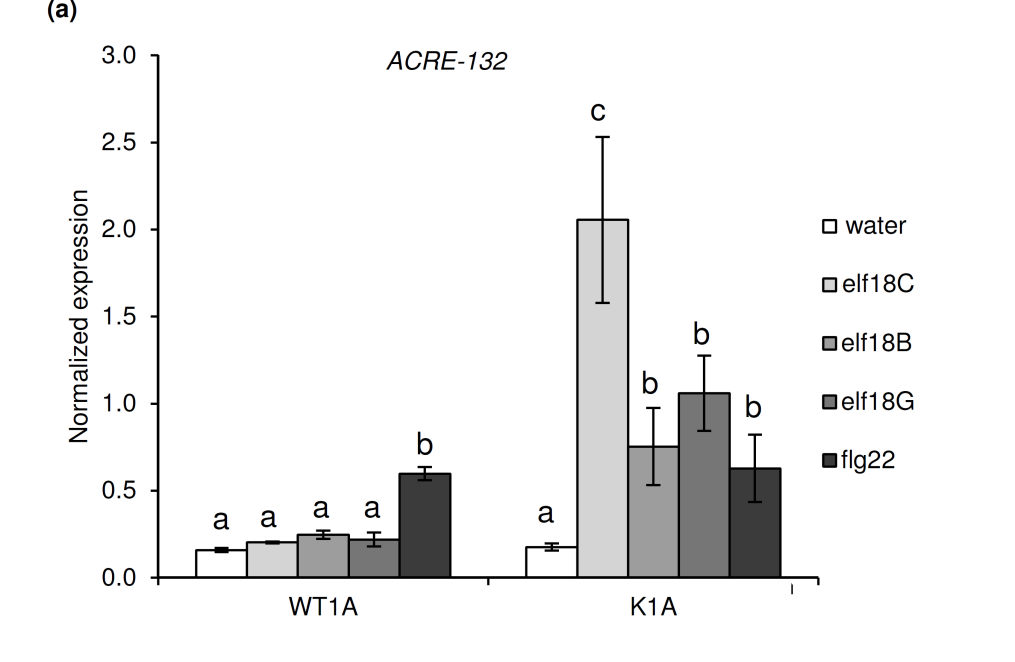


A

B
