# Supplementary material for: An EFR‐Cf‐9 chimera confers enhanced resistance to bacterial pathogens by SOBIR1‐ and BAK1‐dependent recognition of elf18
Source: Mol Plant Pathol. 2019 Apr 1;20(6):751–64. doi: 10.1111/mpp.12789 (PMC6637901; doi:10.1111/mpp.12789)
Supplement: Supplementary file 6 — Fig. S6 Transgenic plants expressing EFR‐Cf‐9 show unaltered susceptibility to Botrytis cinerea. Leaves of soil grown 4‐week‐old wild type (WT) and transgenic plants expressing EFR‐Cf‐9 (K1A and K5A) were inoculated with B. cinerea spore suspension, and lesion areas were measured at 48 hpi. Bars indicate average lesion area ± standard error (SE) (n > 18). No significant difference was observed between WT and transgenic lines (K1A and K5A), according to Student's t‐test (P > 0.5) [file MPP-20-751-s006.docx]

0

2

4

6

8

10

12

WT

K1A

K5A

Lesion area (mm^2^)

**Figure S6. Transgenic plants expressing *EFR-Cf-9* show unaltered susceptibility to *Botrytis cinerea*.** Leaves of soil-grown four-week-old tobacco WT plants and of transgenic plants expressing *EFR-Cf-9* (K1A and K5A) were inoculated with a *B. cinerea* spore suspension. Lesion area was measured 48 h after inoculation. Bars indicate average lesion area ± standard error (n>18). No significant difference was observed between WT and transgenic lines, according to Student’s t-test (p>0.5).
